# *Pseudomonas* Phage PaBG—A Jumbo Member of an Old Parasite Family

**DOI:** 10.3390/v12070721

**Published:** 2020-07-03

**Authors:** Peter Evseev, Nina Sykilinda, Anna Gorshkova, Lidia Kurochkina, Rustam Ziganshin, Valentin Drucker, Konstantin Miroshnikov

**Affiliations:** 1 Shemyakin-Ovchinnikov Institute of Bioorganic Chemistry, Russian Academy of Sciences, 117997 Moscow, Russia; petevseev@gmail.com (P.E.); sykilinda@mail.ru (N.S.); ziganshin@mail.ru (R.Z.); 2 Limnological Institute, Siberian Branch of Russian Academy of Sciences, 664033 Irkutsk, Russia; kovadlo@lin.irk.ru (A.G.); drucker@lin.irk.ru (V.D.); 3 Belozersky Institute of Physico-Chemical Biology, Lomonosov Moscow State University, 119991 Moscow, Russia; lpk56@mail.ru

**Keywords:** *Pseudomonas*, jumbo phage PaBG, bioinformatics, molecular evolution

## Abstract

Bacteriophage PaBG is a jumbo Myoviridae phage isolated from water of Lake Baikal. This phage has limited diffusion ability and thermal stability and infects a narrow range of *Pseudomonas aeruginosa* strains. Therefore, it is hardly suitable for phage therapy applications. However, the analysis of the genome of PaBG presents a number of insights into the evolutionary history of this phage and jumbo phages in general. We suggest that PaBG represents an ancient group distantly related to all known classified families of phages.

## 1. Introduction

Bacteriophages are viruses that infect bacteria and are considered to be a dominant issue for the Earth’s biosphere, with an estimated total of 10^31^ phages on the planet [1,2]. Tailed bacteriophages with DNA genomes above 200 kb are classified as giant or jumbo phages [3]. Such phages have been isolated from diverse environments, including water, soil, marine sediments, plant tissues, silkworms, compost, animal feces and other habitats [3]. The GenBank PHG database contains about 12,000 complete and partial phage genomes as of the end of February, 2020. A small fraction of these genomes (208) can be attributed to jumbo phages. About half of them (103 genomes) are not yet assigned to a specific genus. The hosts of most studied jumbo phages (202) are Gram-negative bacteria, and 17 jumbo phages infect *Pseudomonas*.

Giant bacteriophage PaBG (vB_PaeM_BG according to the recommended unified phage nomenclature [4]) was isolated from a water sample of the ultra-freshwater Lake Baikal [5]. Baikal is the deepest ancient lake in the world, and its autochthonous organic matter has been the subject of numerous studies [6,7]. In particular, a high diversity of bacteriophages in the water of Baikal has been reported [8,9]. PaBG infects *Pseudomonas aeruginosa*, a common inhabitant of freshwater [10]. The phage was propagated in vitro using the classic PAO1 strain of *P. aeruginosa*.

In this work, we analysed the updated data on *Pseudomonas* phage PaBG. We report on phage morphology, host range, details of biological behavior and structural proteomics. The genome of PaBG was re-annotated. It revealed possible details of the evolutionary history of this phage and its relatives. We suggest that the phage represents an ancient group distantly related to all known classified families and report specific genomic characteristics of the phage. We discuss the phage’s evolutionary strategy and its implementation in the phage genome.

## 2. Materials and Methods 

### 2.1. Phage Propagation and Purification

*Pseudomonas aeruginosa* strain PAO1 (ATCC 15692), purchased from the American Type Culture Collection (ATCC), was used as a host for phage propagation. A water sample taken from Lake Baikal (3 mL) was supplemented with 1 mL of 4× lysogeny broth (LB) and 40 µL overnight culture of PAO1, and incubated at 18 °C for 18 h. Chloroform was added to a final concentration of 0.5% (*v*/*v*) for 4 h at 4 °C. The suspension was then centrifuged at 7000× *g* for 20 min. The presence of bacteriophages and the titre in the supernatant was determined by the appearance of plaques on the bacterial lawn using the double-agar layer technique [11] with minor modifications. Soft LB with 0.3% agar was used for the top layer [12]. Ten-fold serial dilutions were prepared from the phage lysates and added to the host bacteria. The mixture was poured onto the bottom agar layer consisting of LB medium. The number of plaques was counted after 18–24 h incubation at 25 °C.

The phage from a single plaque was propagated using a liquid culture of the *P. aeruginosa* PAO1 host strain. The incubation was performed at 25 °C until the lysis was complete, and then chloroform was added. Bacterial debris was pelleted by centrifugation at 7000× *g* for 20 min. The phage lysate was precipitated with polyethylene glycol 8000 (10%)–NaCl (0.6%) at 4 °C overnight, centrifuged at 8000× *g* for 20 min at 4 °C, re-suspended in the SM buffer (50 mM Tris-HCl (pH 7.5), 100 mM NaCl, 8 mM MgSO_4_, 0.01% gelatin) and then 1 M KCl was added. The mixture was incubated on ice for 20 min and centrifuged (12,000× *g* for 20 min at 4 °C) in order to precipitate the PEG solution [13]. The phage preparation was purified by cesium chloride equilibrium gradient centrifugation at 22,000× *g* (Beckman SW41 Ti rotor, Beckman Coulter Inc., Brea, CA, USA) for 2 h. The band with the greatest opalescence was collected and dialysed overnight against 0.01 M Tris–HCl (pH 7.5), 0.01 M MgSO_4_, 0.15 M NaCl at 4 °C. The titre of the resulting phage consisted of about 10^11^ plaque forming units (PFU)/mL.

### 2.2. Phage Biology Experiments

For the adsorption assay, exponentially grown PAO1 cells were mixed with the phage suspension with multiplicity of infection (MOI) = 0.001, and incubated at 25 °C. Samples of 100 µL were taken after 1, 2, 3, 4, 5, 8, 10, 15 and 20 min and then mixed with 850 µL of SM buffer supplemented with 50 µL of chloroform. After centrifugation, the supernatants were titrated for further determination of unabsorbed phages by the plaque assay method [11] at different time intervals. The adsorption constant was calculated according to Adams for a period of 5 min.

For one-step growth experiments, 20 mL of host bacterial cells (OD_600nm_ = 0.3) were harvested by centrifugation (7000× *g*, 20 min, 4 °C) and re-suspended in 0.5 mL LB broth. Bacterial cells were infected with the phage at an MOI of 0.01. The bacteriophage was allowed to adsorb for 5 min at 25 °C. Then, the mixture was centrifuged at 10,000× *g* for 2 min to remove unadsorbed phage particles and the pellet was re-suspended in 20 mL of LB broth. Samples were taken at 5-min intervals over the course of 2 h incubation at 25 °C and immediately titrated.

For the thermal-stability assay, phage suspensions (1 × 10^9^ PFU/mL) were incubated at 40 °C, 50 °C, 60 °C and 70 °C, and aliquots were taken after 20, 40, and 60 min of incubation. For the pH stability assay, a phage suspension (1 × 10^8^ PFU/mL) was inoculated in a series of tubes containing fresh LB broth at pH 4.0, 5.0, 6.0, 7.0, 8.0, 9.0 and 10.0, and incubated at 25 °C; aliquots were taken after 20 h of incubation. Phage titres were determined with *P. aeruginosa* PAO1 as host cells by the double-layer agar method.

The lytic activity and host specificity of phage PaBG were tested against 125 clinical and environmental *P. aeruginosa* strains using the double-layer method with 0.3% soft agar [12]. Briefly, 200 μL of *P. aeruginosa* bacterial cultures grown in LB medium at 37 °C to OD_600_ of 0.4 were mixed with 3 mL of soft agar. The mixture was plated onto the nutrient agar. Then, the phage suspensions (~10^9^ plaque forming units (PFU)/mL) were spotted on the soft agar lawns and incubated at 25 °C for 18–24 h.

All procedures were repeated in triplicate and the results were averaged.

### 2.3. Electron Microscopy

The morphology of phage PaBG was examined by negative strain transmission electron microscopy (TEM) [14]. Briefly, purified and concentrated virus specimens (10^11^ PFU/mL) fixed with 1% glutaraldehyde in 0.1 M phosphate buffer (pH 7.0) were placed on transmission electron microscopy (TEM) support grids, followed by rinsing with distilled water several times. The phage samples were stained with 1% uranyl acetate aqueous solution (pH 4.0) for further examination with a Hitachi H-300TM electron microscope (Hitachi Ltd., Tokyo, Japan). At least 20 phage images were used to assess the particle dimensions.

### 2.4. Structural Proteomics

Structural proteins of phage PaBG purified particles proteins were resolved by 10% SDS–PAGE. Protein bands were excised from the gel and subjected to in-gel trypsin digestion [15]. Peptides were extracted and identified using MALDI-TOF MS (matrix assisted laser desorption ionisation-time of flight mass spectrometry) on an Ultraflex II TOF/TOF mass spectrometer (Bruker Daltonics, Bremen, Germany). The MS data were processed using Bruker Daltonics Flex Analysis 2.4 software (Bruker Daltonics), and the accuracy of mass determination of the peptides was fixed at 100 ppm. The MS data were correlated with the protein sequence using Bruker Daltonics BioTools 3.0 software (Bruker Daltonics).

### 2.5. Reannotation of Phage Genome

The genomic sequence of phage PaBG was downloaded from GenBank (Accession number KF147891) [5] and annotated by predicting and validating open reading frames (ORFs) using Prodigal 2.6.1 [16] and Prokka [17] pipelines. Identified ORFs were manually curated to ensure fidelity. Functions were assigned to ORFs using a BLAST search on a custom phage protein database compiled from annotated phage GenBank sequences, InterPro server (https://www.ebi.ac.uk/interpro/entry/InterPro) and HHpred server (https://toolkit.tuebingen.mpg.de) with Pfam-A_v32.0, NCBI_Consreved_Domain_v.3.16, SMART_v6.0, PRK_6.9, PDB, SCOPe70_2.07, ECOD_ECOD_F70_20190225 and COG_KOG_v1.0 databases. tRNA coding regions were identified with tRNAscan-SE [18] and ARAGORN [19]. The resulting genome map was visualised in Geneious Prime, version 2020.0.5 (https://www.geneious.com).

### 2.6. Phylogenetic Analysis

Bacterial and phage reference genomes were downloaded from NCBI GenBank (ftp://ftp.ncbi.nlm.nih.gov/genbank) and annotated with Prokka [17], with a custom phage protein database compiled from annotated phage GenBank sequences. A search for homologous sequences was conducted using a BLAST search and found sequences were checked for the presence of annotated homologous genes in NCBI genomes. Genes were extracted from GenBank annotations. For some unannotated sequences, ORFs were found by Glimmer [20]. ORFs were validated and corrected by comparison with known homologous genes. Protein alignments were made with MAFFT (L-INS-i algorithm, BLOSUM62 scoring matrix, 1.53 gap open penalty, 0.123 offset value). The alignments were trimmed manually and with trimAL [21] with gappyout settings. Best protein models were found with MEGAX 10.0.5 [22]. Phylograms were generated based on the amino acid sequences of proteins and their concatenated alignments, using Geneious Prime and MAFFT [23] for sequence alignment. Trees were constructed using the maximum likelihood (ML) method with an RAxML program [24] with a WAG+G protein model and the robustness of the trees was assessed by bootstrapping (1000) and with MrBayes [25,26] with a WAG rate matrix, chain length 1,100,000, rate variation gamma, subsampling frequency 200 and unconstrained branch length settings. Heat map analysis was conducted with Gegenees [27] with accurate parameters (fragment length: 200 bp; step size: 100 bp with the threshold set to 5%).

Average nucleotide identity (ANI) was computed using the OrthoANIu tool [28], employing USEARCH (http://www.drive5.com/usearch/) over BLAST (https://www.ezbiocloud.net/tools/orthoaniu) with default settings and with with an EzBioCloud server (https://www.ezbiocloud.net/tools/ani). Genome comparison was made with Easyfig [29]. A protein domain search was conducted with InterPro (http://www.ebi.ac.uk/interpro/). Protein remote homology detection, 3D structure prediction, template-based homology prediction and vizualisation were made by HHpred (https://toolkit.tuebingen.mpg.de/tools/hhpred), Modeller [30], UCSF Chimera [31] and Phyre2 protein fold recognition server [32] (http://www.sbg.bio.ic.ac.uk/~phyre2). Custom BLAST databases were mounted with the BLAST tool (https://blast.ncbi.nlm.nih.gov/). CRISPR loci were checked with the CRISPR Recognition Tool, Version 1.2., by Bland et al. (http://www.room220.com/crt/). Phage defense system loci were obtained from the PADS Arsenal database http://rebase.neb.com/rebase/rebase.html.

Genome-based phylogeny was obtained using the VICTOR server (http://ggdc.dsmz.de/). All pairwise comparisons of the nucleotide sequences were conducted using the Genome-BLAST Distance Phylogeny (GBDP) method [33] under settings recommended for prokaryotic viruses [34]. The resulting intergenomic distances were used to infer a balanced minimum evolution tree with branch support via FASTME, including SPR postprocessing [35] for each of the formulas D0, D4 and D6. Branch support was inferred from 100 pseudo-bootstrap replicates each. Trees were rooted at the midpoint [36] and visualised with FigTree (http://tree.bio.ed.ac.uk/software/figtree/). The phage proteomic tree was obtained using the ViPTree server (https://www.genome.jp/viptree/) [37].

## 3. Results

### 3.1. General Biological Characteristics of Phage PaBG

**Figure 1:**
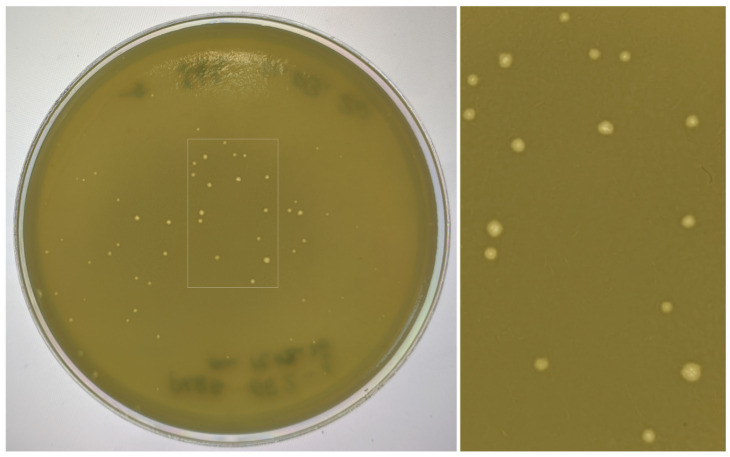
Plaques with opaque haloes formed by phage PaBG on the lawn of *Pseudomonas aeruginosa* PAO1.

The jumbo bacteriophage PaBG was isolated from a water sample from Lake Baikal in 2010 [5]. When using a standard double-layer agar protocol [11], the phage formed tiny pinhole-sized plaques on the lawns of susceptible *P. aeruginosa* strains. Plaque formation occurred at ambient temperatures (18 °C–30 °C), but not at 35 °C and above. When the concentration of the top agar was reduced to 0.3% to promote the diffusion of the phage [12], the plaques became more pronounced, resulting in ~1 mm in size with a surrounding halo (Figure 1). The haloes expanded in size during the subsequent storage of the Petri dish at room temperature, while lysis zones of clear plaques remained constant. The production of such haloes is indicative of phage-induced degradation of bacterial exopolysaccharides [38].

**Figure 2:**
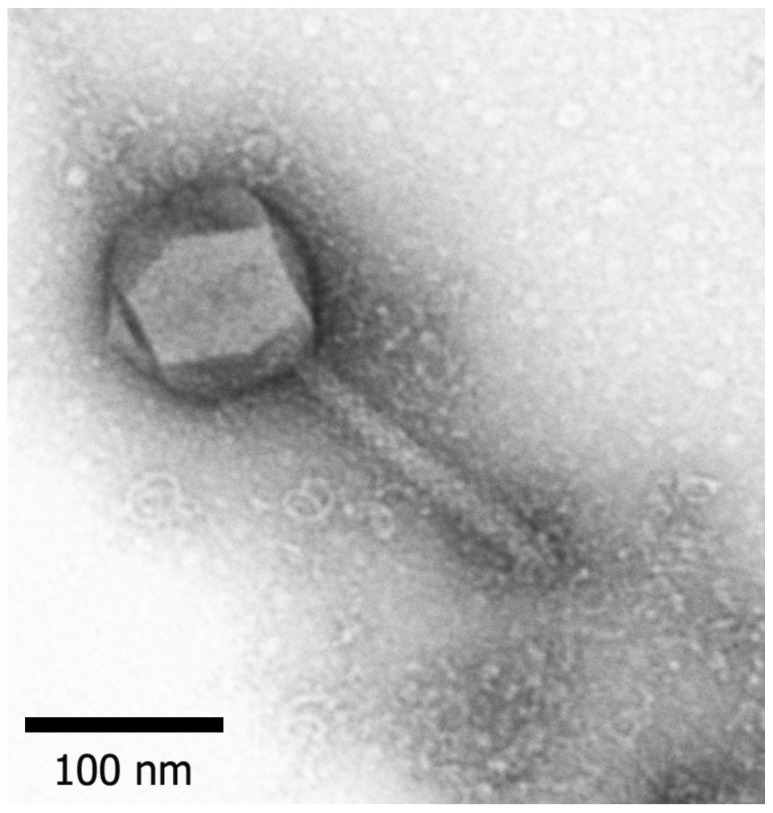
Electron microscopy image of phage PaBG. Staining with 1% uranyl acetate. Scale bar—100 nm.

The morphology of phage PaBG revealed by TEM (Figure 2) is typical for myovirus group A1 [39]. Phage PaBG had an icosahedral head 136 ± 8 nm in diameter and a contractile tail 220 ± 12 nm long and 20 ± 3 nm wide. Small appendages comprising the adsorption apparatus of the phage were observed on the end of the tail.

**Figure 3:**
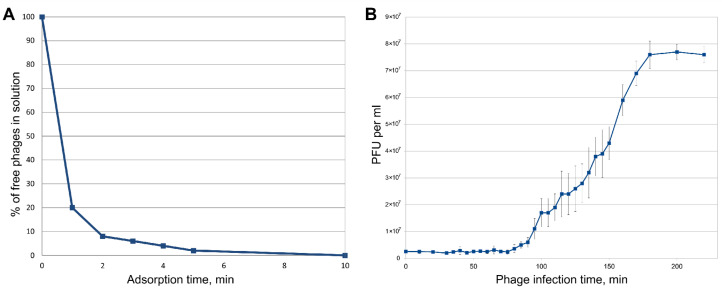
(**A**) Adsorption of phage PaBG to host bacteria. (**B**) One step growth curve of PaBG using *P. aeruginosa* PAO1 as a host.

Infectivity assays showed fast adsorption of the phage. As shown in Figure 3A, more than 95% of PaBG particles adsorbed to the host cells within 5 min at 25 °C. The estimated adsorption constant was 1.0 × 10^−8^ mL/min. The one-step growth curve shows a latent period of 80 ± 5 min, slow lysis and a moderate burst size of 30–40 PFU/cell at 25 °C (Figure 3B).

The infection range of phage PaBG was relatively narrow. Of 125 assayed field and clinical isolates of *P. aeruginosa*, only 20 were susceptible to PaBG. These strains were not characterised comprehensively, therefore we were unable to assess their genetic and phenotypic diversity (Supplementary Table S1).

The phage did not lose activity in the ambient range of pH 5–9 at room temperature. However, PaBG was thermally sensitive, retaining only 5% activity after 60 min of incubation at 50 °C, and completely losing infectivity after 5 min of heating to 60 °C.

### 3.2. Structural Proteome of Phage PaBG

**Figure 4:**
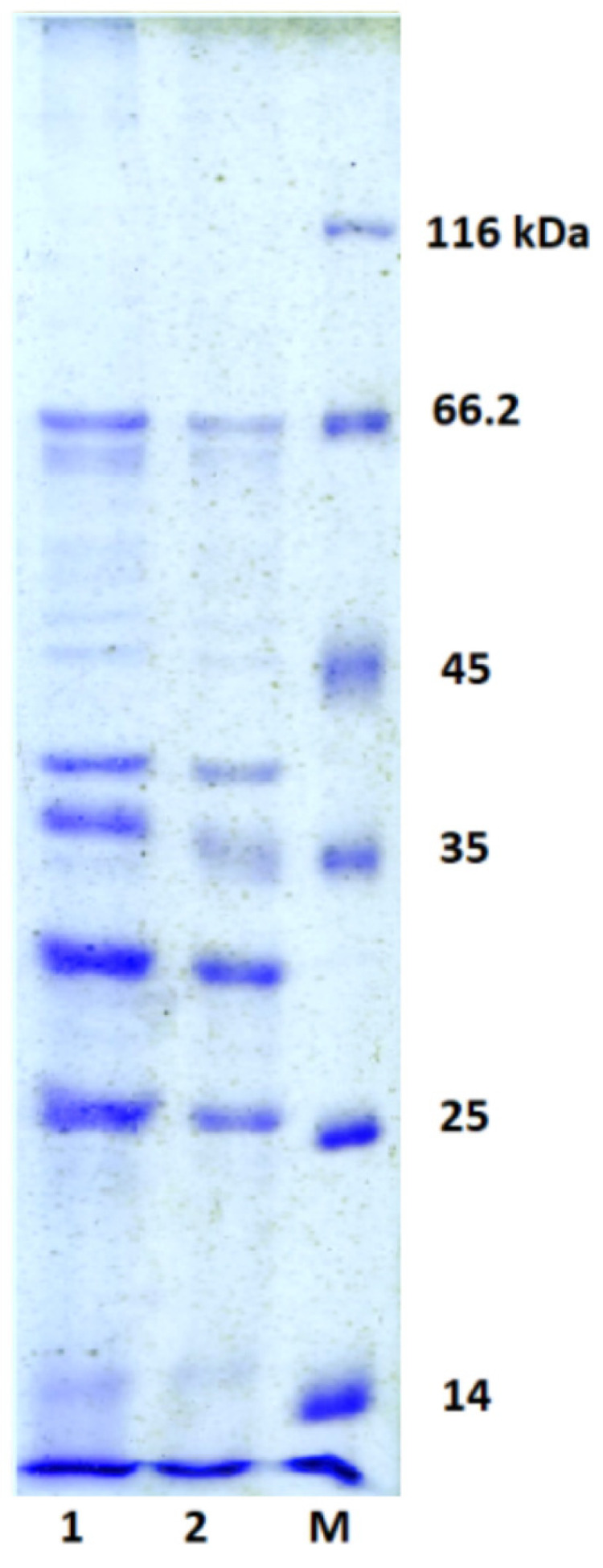
Ten percent SDS-PAGE analysis of the phage PaBG structural proteins. 20 µL (1) and 10 µL (2) of protein solution were applied to gel. M—protein molecular weight marker.

The pattern of the PaBG structural proteins is shown in Figure 4. Denatured PaBG particles were separated by SDS–PAGE after purification by ultracentrifugation in the CsCl gradient. We were able to identify 14 proteins present in the concentration sufficient for detection by Coomassie Blue staining (Table 1). Comparison of tryptic maps and predicted translated sequences shows that, similar to the other large phages, some PaBG virion proteins were subject to proteolytic processing [40]. The molecular weight of PaBG phage major capsid protein decreased by about 20% upon maturation. Polypeptides matching gp80, gp81, gp191, gp213 and gp215 were identified in multiple SDS-gel bands, so the corresponding proteins apparently contained multiple sites of proteolysis. In addition to expected structural proteins (Table 1), a number of PaBG gene products, mostly with an unknown function, were identified as virion-associated proteins.

### 3.3. PaBG Genome—General Features

**Figure 5:**
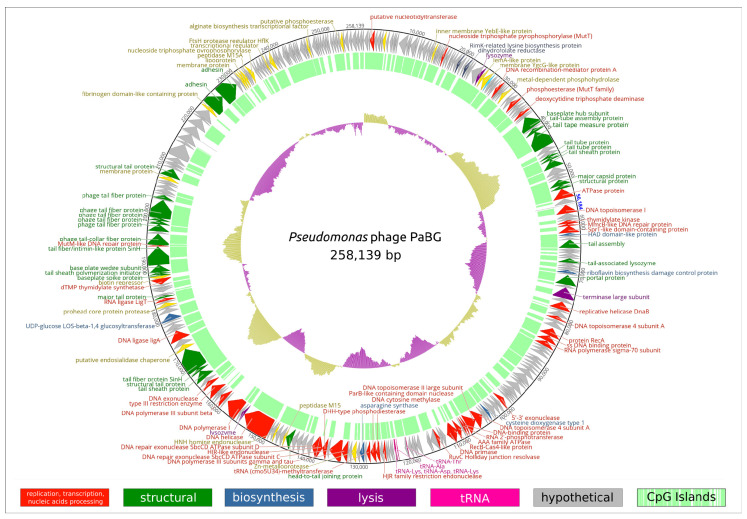
Circular diagram of functional assignments of the *Pseudomonas* phage PaBG genome. In total, 101 protein-coding genes and five tRNA genes are shown as coloured blocks. Hypothetical proteins are coloured grey. The direction of transcription is shown by arrows. CpG islands are indicated by the internal green histogram. The GC content of the genome sequence is indicated by the internal purple or yellow histograms. Full annotations and the GenBank file can be found in Supplementary File S1 and Supplementary Table S2.

The genome of phage PaBG [5] was re-annotated, exploiting the data on recently studied jumbo phage genomes and protein structures. The length of the PaBG dsDNA genome is 258,139 bp (accession number KF147891) and, similar to most jumbo phages, it exists in a circular permuted form. The genome (Figure 5) contains 317 putative ORFs, putative functions of 101 proteins can be predicted, and 216 ORFs are assigned as hypothetical proteins (Supplementary Table S2). There are five tRNA genes found in the genome. The genes are clustered in two cascades oriented in opposite directions. A block of 112 genes is oriented clockwise and another block of 205 genes is oriented anti-clockwise. The phage appears to possess a sophisticated system of DNA replication and processing, which includes DNA polymerases I and III, DNA ligases and topoisomerases, Holliday junction resolvases, ParB-like nuclease, etc. A BLASTP search of translated predicted coding sequences on the NCBI nr/nt database identified that the homologs for most of the PaBG gene products are of bacterial origin. A significant part of PaBG predicted proteins is more similar to representatives of other kingdoms of life than to viruses. Several proteins show similarity to ribosomal proteins. Several genes encode nucleic acid and amino acid metabolism proteins. Of the structural proteins of PaBG, the tail sheath consisting of two separate proteins should be noted [41].

### 3.4. Taxonomy

**Figure 6:**
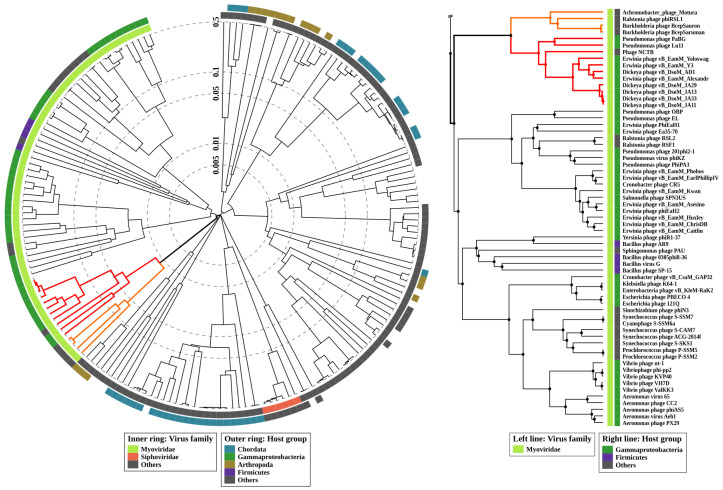
Circular proteomic tree of 184 phages and non-bacterial dsDNA viruses with genome size above 200,000 (**left**), and the *Myoviridae* part of the tree (**right**) constructed using ViPTree. The branches representing the phages of the Lupandier group (*Pseudomonas* phage Lu11, *Pseudomonas* phage PaBG, Phage NCTB, *Dickeya* phages vB_DsoM_AD1, vB_DsoM_JA13, vB_DsoM_JA29, vB_DsoM_JA11, vB_DsoM_JA33 and *Erwinia* phages vB_EamM_Y3, vB_EamM_Yoloswag, vB_EamM_Alexandra) are coloured red, and the branches representing the *Mieseafarmvirus* phages are coloured orange.

**Figure 7:**
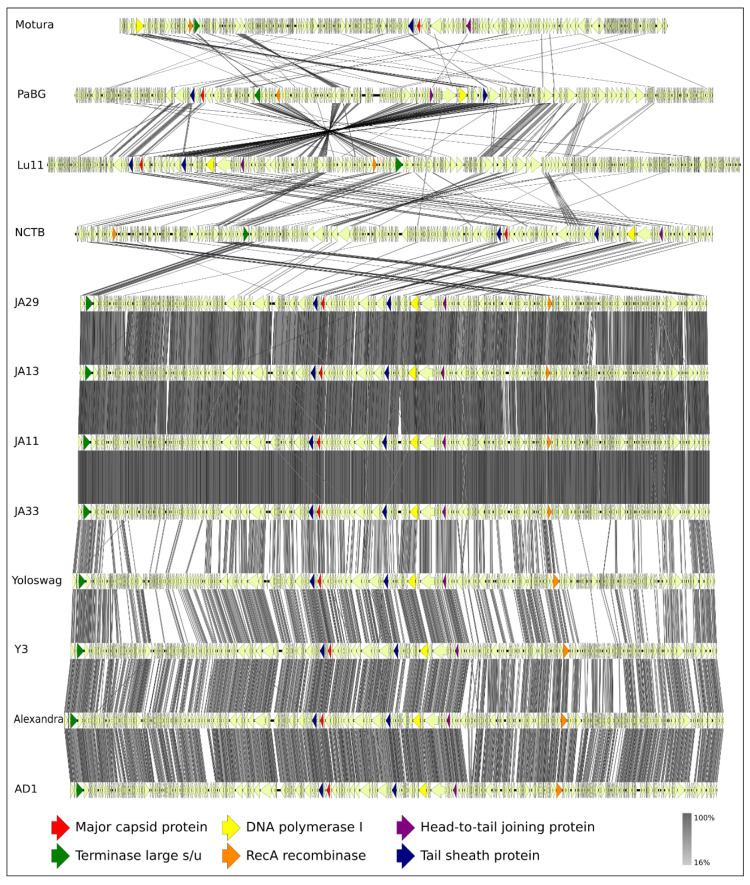
Genome sequence comparison among 12 PaBG-related viral genomes exhibiting co-linearity detected by TBLASTX. Phage abbreviations are as follows: Motura, *Achromobacter* phage Motura (*Mieseafarmvirus*); PaBG, *Pseudomonas* phage PaBG; Lu11, *Pseudomonas* phage Lu11; NCTB, Phage NCTB; JA29, *Dickeya* phage vB_DsoM_JA29; JA13, *Dickeya* phage vB_DsoM_JA13; JA11, *Dickeya* phage vB_DsoM_JA11; JA33, *Dickeya* phage vB_DsoM_JA33; Yoloswag, *Erwinia* phage vB_EamM_Yoloswag; Y3, *Erwinia* phage vB_EamM_Y3; Alexandra, *Erwinia* phage vB_EamM_Alexandra; AD1, *Dickeya* phage vB_DsoM_AD1. The percentage of sequence similarity is indicated by the intensity of the grey colour. Vertical blocks between analysed sequences indicate regions with at least 16% similarity.

Calculations of average nucleotide identity (ANI) between PaBG and all 12,207 phage genomes deposited in the NCBI GenBank (up to February 2020) with OrthoANIu revealed 11 phages with genomic ANI 58.4%–63.0% compared to PaBG (Supplementary Table S3), which can be considered as distant relatives to phage PaBG. The genome with the greatest similarity (63%) belongs to the jumbo phage Lu11, infecting *Pseudomonas putida* [42]. Other phages found with OrthoANIu were cyanobacterial *Trichodesmium* phage NCTB (NCBI Accession number LT598654) [43] with ANI 59.0%, *Achromobacter* phage Motura (Accession number MN094788) and several phages infecting *Dickeya* (vB_DsoM_AD1—MH460463, vB_DsoM_JA13—MH460460, vB_DsoM_JA29—MH460461, vB_DsoM_JA11—MH389777, vB_DsoM_JA33—MH460462) [44] and *Erwinia* (vB_EamM_Y3—KY984068, vB_EamM_Yoloswag—KY448244, vB_EamM_Alexandra—MH248138) [45,46]. *Achromobacter* phage Motura was attributed to the *Mieseafarmvirus* genus, while the other phages belong to unclassified groups. Proteomic (Figure 6) and full-genome (Supplementary Figure S1) phylogeny supported the placement of PaBG and the phages listed above in the same unclassified group, (we refer to this group of phages as the “Lupandier group”—Lu11, PaBG, NCTB, *Dickeya* and *Erwinia* phages). The genome comparison made with TBLASTX (Figure 7) corresponds to full-genome phylogeny and shows genome rearrangements accompanying the phage evolution, and the distinct outlying position of PaBG, Lu11 and NCTB, compared to the phages of *Dickeya* and *Erwinia*.

**Figure 8:**
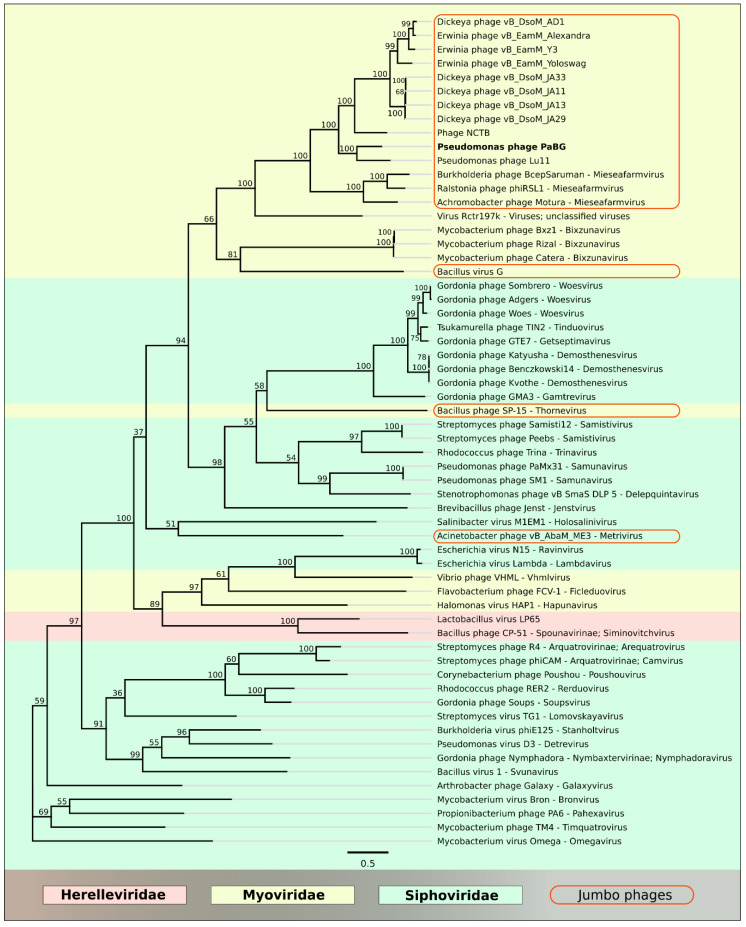
Best-scoring tree found by maximum likelihood (ML) search with RAxML based on the major capsid protein and terminase large subunit concatenated protein sequences. Taxonomic classification was taken from NCBI sequence attributes and is shown to the right of the organism name. Bootstrap support values are shown above their branch as a percentage of 1000 replicates. The scale bar shows 0.5 estimated substitutions per site and the tree was unrooted.

The major capsid protein (MCP) and terminase are the two most conserved proteins encoded in bacteriophage genomes, and they have been frequently used for taxonomic grouping of phages [47], including jumbo phages [48]. In order to construct a consistent taxonomy and phylogenetic positioning of phage PaBG, we performed a BLAST search using the GenBank phage database, and created a list of 350 phages belonging to the taxa, representatives of which were found with E-value < 10^−3^. We also added the phages presenting other taxa to the list and extracted the genes of major capsid protein (MCP) and a large subunit of terminase. The ML phylogeny of MCP and terminase protein sequences showed an undefined putative evolutionary history for these proteins (Supplementary Figures S2 and S3). However, the closest neighbours of PaBG formed a similar list including the members of the *Mieseafarmvirus* genus and an unclassified Virus Rctr197k from an anaerobic methane oxidising methylomirabilis bioreactor enrichment culture with the sequenced partial genome of 197 kb [49]. We also constructed an RAxML phylogenetic tree with 350 concatenated protein sequences of MCP and terminase (Supplementary Figure S4), to find PaBG relatives more confidently. The trees place the Lupandier group separately from other jumbo phage groups, and distantly from *Phikzviruses*, *Agricanviruses*, *Machinaviruses*, *Seoulviruses* and *Asteriusviruses*. Utilising sequences in the branches adjacent to PaBG clades on the 350-sequence tree (Supplementary Figure S4), we constructed a phylogenetic tree of protein sequences of concatenated major capsid protein and a large subunit of terminase with higher bootstrap-support comprising 60 phage genomes (Figure 8). The tree also groups 11 phages (PaBG, Lu11, phage NCTB, vB_DsoM_AD1, vB_DsoM_JA13, vB_DsoM_JA29, vB_DsoM_JA11, vB_DsoM_JA33, vB_EamM_Y3, vB_EamM_Yoloswag and vB_EamM_Alexandra) into a distinct clade and points to *Mieseafarmvirus* as the closest genus.

A BLASTP search using the PaBG major capsid protein sequence, NCBI environmental sequences and metagenome proteins databases revealed homologous sequences which can represent bacteriophages belonging to the PaBG group. Phylogenetic trees with environmental and metagenome sequences are shown in Supplementary Figure S5. The size of some of the environmental sample sequences exceeds 100,000 bases. Sequences CENZ01040713 and CEPA01131689 (with E-value 6.4 × 10^−109^) are even longer than 200 kb (probably presenting undescribed jumbo phages). A BLAST search of predicted proteins showed that they were very close to phage NCTB and could be members of the Lupandier group. We also conducted a TBLASTN search of the PaBG predicted protein sequences using a custom database constructed with 375 published phage-like and plasmid-like sequences. These sequences were acquired from metagenomic datasets from human fecal and oral samples, fecal samples from other animals, freshwater lakes and rivers, marine ecosystems, sediments, hot springs, soils, deep subsurface habitats and the built environment [50], and we found homologous sequences (BitScore > 50) for 111 PaBG predicted proteins. This suggests that PaBG-like phages are more widespread in different ecological niches than it seems.

### 3.5. Particular Features of PaBG Genes

The current understanding of phage genomics and proteomics enables us to suggest possible functions for only 101 predicted proteins out of 317 (Supplementary Table S2). A BLASTP protein search with an NCBI non-redundant (nr) database and E-value < 10 cannot recognise even remote homologs in all known organisms for 10 predicted proteins, so these proteins can be considered unique for PaBG. A BLASTP protein search with an NCBI non-redundant (nr) database and E-value < 10^−5^ recognises homologs for only 167 predicted proteins, and 47 of them belong to non-viral organisms. A search with a higher E-value cut-off (<0.1) yields results for 200 proteins, and 67 of them belong to organisms of kingdoms other than those of viruses. Surprisingly, most predicted genes were found to have homologous genes in the genome of *Salmonella enterica* subsp. *enterica* serovar Infantis strain 159669 (NCBI AAHPOS010000001). This sequence was assigned as a contig of the *Salmonella enterica* subsp. *enterica* serovar Infantis whole genome shotgun sequence in 2018, but it seems more plausible that it presents a nearly complete genome of a phage belonging to the Lupandier group, judging by a TBLASTX comparison (Supplementary Figure S6). Calculations of ANI of *Salmonella enterica* subsp. *enterica* serovar Infantis sequence fragment AAHPOS010000001 with EzBioCloud server (https://www.ezbiocloud.net/tools/ani) pointed to *Dickeya* phages vB_DsoM_AD1 as a possible closest relative (77.05% similarity) and 59.1% genome similarity when compared to PaBG.

At least several predicted proteins encoded in the PaBG genome can function as damage response proteins. They can include nucleoside triphosphate pyrophosphorylase (*mutT*, gene 30), N-glycosylase of 5-amino-6-ribosylamino-2,4-pyrimidinedione 5′-phosphate (riboflavin biosynthesis damage control, gene 104), DNA repair exonuclease ATPase subunit C (*sbcC*, gene 186), 185-DNA repair exonuclease ATPase subunit D (*sbsD*, gene 191), ATP-dependent DNA repair recombination helicase, that can function also as components of a phage’s own DNA restriction-modification mechanisms (*uvsW,* gene 210), and MutM-like DNA repair protein (gene 242). Several predicted proteins can be involved in amino acid, nucleic acid and other metabolism processes, including RimK-related lysine biosynthesis protein (gene 37), dihydrofolate reductase (gene 40), deoxycytidine triphosphate deaminase (gene 65), thymidylate kinase (gene 87), putative cysteine dioxygenase type 1 (gene 143), asparagine synthase (gene 180), DHH-type phosphodiesterase (gene 189), UDP-glucose LOS-beta-1,4 glucosyltransferase (gene 223), dTMP thymidylate synthase (gene 236) and alginate and motility regulator (*algZ*, gene 303). Interestingly, alginate and motility regulator AlgZ can be involved in the processes of biofilm formation in *Pseudomonas aeruginosa* [51].

The PaBG genome contains five tRNA genes, including tRNA-Ala, tRNA-Asp, tRNA-Thr and two tRNA-Lys. The genome also contains genes encoding protein similar to RecB-like nuclease Cas4 [52], but no corresponding CRISPR loci were identified.

#### 3.5.1. DNA and RNA Polymerases

Genes of the polymerases of nucleic acids are usually readily recognisable by a similarity search. The genome of PaBG contains putative genes encoding both DNA and RNA polymerases. DNA polymerases are represented with the genes of multi-domain DNA polymerase I complex, including Klenow fragment (gene 205) and 5′→3′ exonuclease DNA polymerase subunit (gene 137), DNA polymerase III γ subunit (gene 207) and DNA polymerase III β subunit (gene 185). The structure of DNA polymerase I (gene product, gp 205), predicted by analysis of the sequence with protein databases, HHpred search and homologous modelling, includes the N-terminal uracil DNA glycosylase domain, the following 3′→5′ exonuclease domain and the C-terminal 5′→3′ polymerase domain. Analysis of alignments and HHpred server search confirmed the presence of these three domains in all 11 members of the Lupandier group, but a BLASTP search did not find sequences close to PaBG uracil DNA glycosylase among phage sequences presented in the nr NCBI database beyond the group (E-value < 10).

The uracil DNA glycosylase domain can eliminate uracil residues from DNA molecules by cleaving the N-glycosidic bond and initiating the base excision repair pathway [53]. Uracil is one of the most frequently occurring erroneous bases in DNA; it can arise from cytosine deamination or thymine-replacing incorporation and can increase mutagenesis rate. Thus, uracil DNA glycosylase is important for genome stability maintenance [54]. Some bacteriophages are tolerant to the partial incorporation of uracil bases to the genome that can have an evolutionary impact by increasing genetic variation [55,56].

Interestingly, the phylogenetic tree of DNA polymerase I (gene 205) built with sequences found by BLAST search in the nr NCBI database and custom databases points to the DNA polymerase I of the *Thermoplasmata* archaeon (*Archaea*; *Euryarchaeota*; *Diaforarchaea* group) as the closest relative, which possesses common ancestry with the Lupandier group. We carried out phylogeny with both trimmed and full alignments, and with different algorithms (MrBayes, RAxML, FastTree), protein models (WAG, WAG I+F, LG+G+I, BLOSUM62), settings and outgroups, and the topology of the trees was very close, supporting the placement of the *Thermoplasmata* archaeon. The contig of *Thermoplasmata* archaeon genome assembly from the metagenome data, containing the DNAP I gene, also contained homologs for the major capsid protein and terminase genes. It is possible that this sequence also presents a phage. The phylogenetic tree of DNAP I from 100 organisms is shown in Supplementary Figure S7. The predicted 3D structure of DNAP I obtained by Phyre2 homologous modelling is shown in Supplementary Figure S8.

Another protein of PaBG encoded by gene 137, with a sequence similar to that of DNA polymerase I, supposedly provided a 5′→3′ exonuclease activity. An analysis of its amino acid sequence with protein databases suggested the presence of an N-terminal PIN domain and a C-terminal 5′→3′ exonuclease domain. Well studied 5′→3′ exonucleases of phage origin include bacteriophage T4 RNase H, which has a sequence similar to the RAD2 family of eukaryotic proteins [57], and bacteriophage T5 5′-exonuclease [58]. PIN-domains cleave single-stranded RNA in a sequence-specific, Mg^2+^- or Mn^2+^-dependent manner, and in prokaryotes they are found to be the toxic components of toxin-antitoxin (TA) systems, their toxicity arising by virtue of their ribonuclease activity [59]. We hypothesised that PaBG 5′→3′ exonuclease (gp 137) provides both RNA–DNA and DNA–DNA exonuclease activity, and participates in replication removing DNA and RNA primers from the lagging strand of DNA to allow Okazaki fragments to bind to, and work together with, the Klenow fragment (gp 205). The phylogenetic tree of 70 sequences homologous to 5′→3′ exonuclease is shown in Supplementary Figure S9. Most phage sequences are grouped in a single large clade neighbouring the eukaryotic homologs. The predicted 3D structure of a 5′→3′ exonuclease domain of DNA polymerase I obtained by Phyre2 homologous modelling is shown in Supplementary Figure S8. The predicted topology of the protein is similar to the known structures of 5′→3′ exonucleases.

In addition to predicted DNA polymerase I genes, the PaBG genome contained genes for DNA polymerase III subunit γ (gene 185) and DNA polymerase III subunit β (gene 207). The γ and τ subunits of DNA polymerase III holoenzyme are both products of the *dna*X gene. They are homologous to clamp-loading proteins of many organisms, from phages to humans [60]. The full-length product of the *dna*X gene in *Escherichia coli* encodes the DNA polymerase III τ subunit [61]. A translational frameshift leads to early termination and a truncated protein subunit γ, about one third shorter than τ and present in roughly equal amounts. The length of the predicted gene of DNA polymerase III subunit γ (gene 180) is 68.7% of the length of the *dnaX* gene of *Thermus thermophilus* (strain JL-18) and 59.0% of the length of gene encoding γ and τ subunits in *Escherichia coli* (strain CI5). The nucleotide alignment demonstrates that the PaBG gene is homologous to the 5′ end of both *E. coli* and *T. thermophilus* genes. We might suggest that the PaBG gene encodes only the γ subunit. Three dimensional structure Phyre2 homology modelling aligned 91% of the PaBG γ subunit predicted protein sequence with *E. coli* DNA polymerase III subunit γ, with 91% coverage and 100% confidence (Supplementary Figure S10-II).

**Figure 9:**
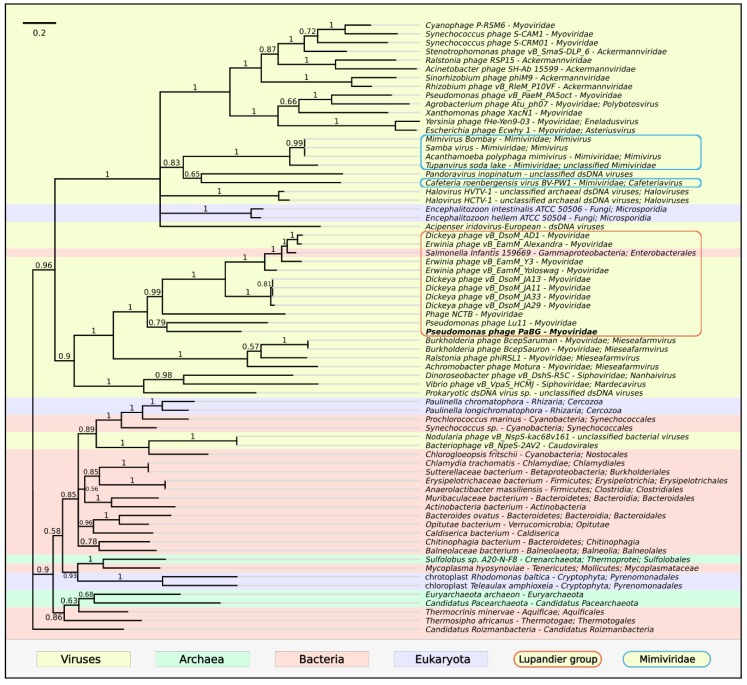
Phylogenetic tree obtained with MrBayes based on DNA polymerase III subunit γ protein sequences and homologous sequences found with a BLAST search of NCBI databases. Bayesian posterior probabilities are indicated above their branch. Taxonomic classification is taken from NCBI sequence attributes and is shown to the right of the organism name. The scale bar shows 0.2 estimated substitutions per site and the tree was rooted to *Candidatus Roizmanbacteria*; 3,000,000 generations sampled every 100 generations and an average standard deviation of split frequencies of 0.0103.

The phylogenetic tree of 70 protein sequences homologous to PaBG DNA polymerase III subunit γ, found with a BLAST search of different organisms, is shown in Figure 9. The tree places jumbo phages in two distinct adjacent clades; one of them includes phages of the Lupandier group, *Mieseafarmvirus* and others, and the other clade includes a number of Myoviridae jumbo phages, Ackermannviridae phages, Mimiviridae viruses and Microsporidia. Interestingly, DNAP III subunit γ homologous sequences with distant similarity were found with a BLAST search of the GenBank viral database in ssRNA Caliciviridae viruses polyprotein gene of Wuhan spiny eel calicivirus 2 (E-value 1.26 × 10^−2^, BitScore 44.7), Mink calicivirus (E-value 1.71 × 10^−2^, BitScore 44.2) and other Caliciviridae viruses.

A search of homologs of predicted PaBG DNA polymerase III subunit β with BLASTP on nr NCBI database (E-value < 10^−5^) revealed similar sequences only in all phages of the PaBG group and some bacteria. Phylogenetic analysis pointed to Thermotogae as a sister clade (Supplementary Figure S11). The phylogenetic tree reliably groups Lupandier phages as a distinct clade, neighbouring genus *Mieseafarmvirus.* The predicted 3D structure of DNA polymerase III subunit β obtained by Phyre2 homologous modelling is shown in Supplementary Figure S10-I.

The PaBG genome contains at least one RNA polymerase σ^70^ factor gene (*rpoD*, gene 118). The sigma factor, which reversibly associates with the core RNA polymerase complex to form a holoenzyme, is required for transcription initiation from promoter elements. RNA polymerase recruits alternative sigma factors as a means of switching on specific regulons [62]. The primary protein sequence of the PaBG σ^70^ factor encoded by gene 118 has no significant similarity with other known sequences outside the Lupandier group and *Mieseafarmvirus*. However, the presence of specific motifs for the σ^70^ factor and a search involving homology detection and structure prediction by hidden Markov model HMM-HMM comparison (HHpred: probability 99.92%, E-value 2.8 × 10^−22^) enables the function to be predicted with a fair level of confidence. The phylogenetic tree of PaBG RNA polymerase σ^70^ factor (gene 118) is shown in Supplementary Figure S12. It groups the Lupandier phages as a separate clade adjacent to *Mieseafarmvirus*. There is another gene in the PaBG genome, possibly encoding putative sigma factor (gene 112), but one cannot be confident of its function because of the weak similarity of its structure to known proteins (HHpred: probability 97.2%, E-value 0.2). The structure of the protein encoded by gene 112 shows some similarity with the SigmaE factor (HHpred: probability 96.87%, E-value 0.28) that controls the extracytoplasmic stress response in *Escherichia coli* [63].

#### 3.5.2. Major Capsid Protein

Phage PaBG’s predicted major capsid protein, encoded by gene 80, belongs to HK97-like fold-containing MCPs [64]. The amino acid sequence of the PaBG MCP shares a few similarities with major capsid proteins of other phages outside the PaBG group and *Mieseafarmvirus*, except for Virus Rctr197k and Bacillus virus G. However, structure prediction by HMM-HMM indicated structural similarity to a major capsid protein of *Propionibacterium* phage PA6 (HHpred: probability 99.9%, E-value 2.9 × 10^−22^), *Enterobacteria* phage HK97 (HHpred: probability 99.9%, E-value 1.3 × 10^−22^), *Escherichia* phage T5 (HHpred: probability 99.89%, E-value 2.8 × 10^−22^) and others. The predicted 3D structure of PaBG MCP obtained by Phyre2 homologous modelling is shown in Supplementary Figure S13. Phylogenetic analysis (Supplementary Figure S14) confirmed the relations with the above mentioned *Thermoplasmata* archaeon, as in the case of DNA polymerase I.

#### 3.5.3. Terminase

The terminase large subunit was predicted to be a product of gene 108. Phylogenetic analysis demonstrated that Lupandier and *Mieseafarmvirus* phage terminases share a common ancestry with proteins from *Ewingella americana* Gammaproteobacterium and *Thermoplasmata* archaeon metagenome assembly (Supplementary Figure S15). The phylogeny of terminase, as well as of MCP, points to *Mycobacterium* phages of *Bixzunavirus* genus as possible predecessors of diverse groups of jumbo phages belonging to *Myoviridae* and *Siphoviridae.* It is worth noting that alignments and a protein database search of predicted terminase large subunits pointed to an intein insertion in a number of Lupandier phages (Supplementary Figure S16). Inteins were shown to participate in phage terminase evolution [65,66].

#### 3.5.4. Tail Sheath Proteins

**Figure 10:**
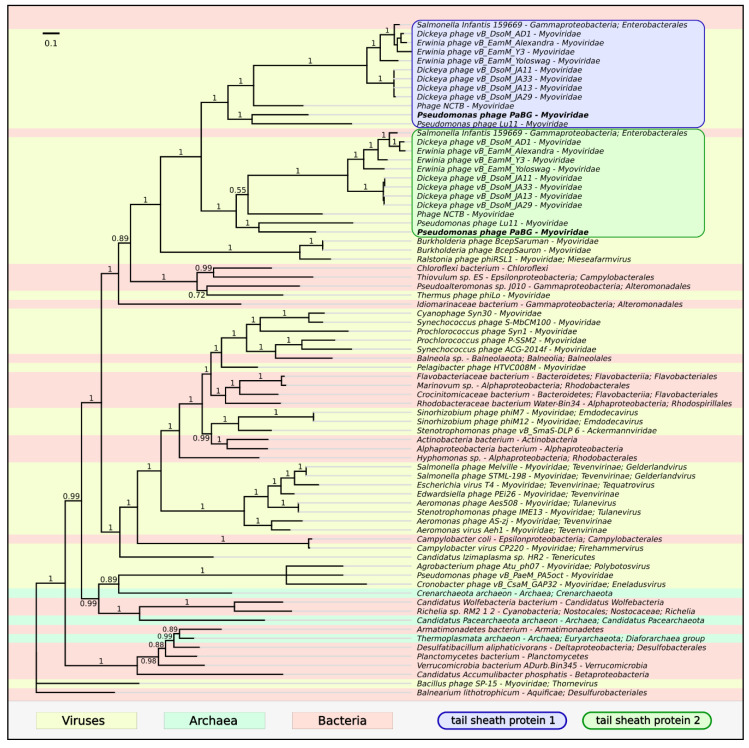
Phylogenetic tree obtained with MrBayes based on tail sheath protein sequences and homologous sequences found with a BLAST search of NCBI databases. Bayesian posterior probabilities are indicated above their branch. Taxonomic classification is taken from NCBI sequence attributes and is shown to the right of the organism name. The scale bar shows 0.1 estimated substitutions per site and the tree was rooted to *Bacillus* phage SP-15; 1,100,000 generations sampled every 200 generations and an average standard deviation of split frequencies of 0.0036.

The giant *Pseudomonas aeruginosa* phage PaBG is notable for its possession of two tail sheath proteins [28]. This is a trait that distinguishes putative members of the suggested Lupandier group from evolutionarily close *Mieseafarmvirus* phages. The proteins shared only 38% sequence identity, but, according to the bioinformatics analysis, the spatial structure of both proteins was similar. Phylogenetic analysis made it possible to suggest that they have a common predecessor and have arisen by gene duplication after the divergence of *Mieseafarmvirus* and Lupandier groups (Figure 10).

## 4. Discussion

Jumbo phages described to date were shown to have a complex evolutionary origin and high diversity, including over 11 clusters and five singleton bacteriophages suggested from 52 complete jumbo bacteriophage genomes analysed in 2017, many of which are uncharacterised [3,48,67,68].

As demonstrated by phylogenetic analysis of conservative proteins, as well as by means of whole-genome proteomic and nucleotide phylogeny, the phages studied in this research (members of the so-called “Lupandier group”) present a distinct monophyletic clade, comprising jumbo phages infecting taxonomically distant hosts. Moreover, the analysis of sequences from open databases demonstrated that the “Lupandier phages” may be members of a larger taxonomic group, which also includes phages of genus *Mieseafarmvirus* and uncultured phages from different habitats not yet described in detail. Lupandier phages possess a number of common genomic features. These features include subunit β of DNA polymerase III, which is distant in evolutionary terms from other phage polymerases, and unusual DNA polymerase I, containing uracil-DNA glycosylase domain. Phylogenetic analysis of DNA polymerase III subunit γ pointed to the attachment (together with some phages) to one of two big clades, with the other clade having included eukaryotic and archaeal viruses, bacteriophages (basically *Siphoviridae*) and eukaryotes. RNA polymerase σ^70^ factor differs from the sequences of such factors in other phages, except for *Mieseafarmviruses.* Another specific feature of the Lupandier group is the presence of two tail sheath proteins, which, as was shown by phylogenetic analysis, arose by gene duplication. On the other hand, “Lupandier” phages share common traits with other jumbo phages, including a high proportion (about 70%) of orphan genes, advanced replication and transcription apparatus [3,69], multiple structural proteins [70] and the possession of homologs for many genes with predicted functions among other jumbo phage genomes.

Recent analysis of metagenomic data [50,71,72,73] indicates the widespread presence of jumbo phages in nature. Hundreds of new sequences, from various metagenome sources, potentially belonging to jumbo phages have been found [48], and our preliminary analysis showed that some of them can belong to the Lupandier group. The detailed taxonomy of jumbo phages needs focused studies. Our results suggest that the Lupandier group and phages, related in an evolutionary context, could also be found in different environments such as fresh and marine water, anaerobic methane-oxidising microbial communities, the human gut and plant pathogens.

This prevalence and diversity extend the list of questions about the features of evolutionary strategy and implementation of these features in the genomes of phages including a Lupandier group. Different parasites have different strategies and can adhere to a strategy during their long evolutionary pathway, since a change in strategy can cause a temporary reduction in fitness. There is no single successful strategy, otherwise Darwinian demons would exist in the real world. Viruses also have different evolutionary strategies that are reflected in their lifestyle [74].

The evolution of phages could be described with the help of the Red Queen Hypothesis (RQH) [75]. This is a hypothesis originally developed for palaeontology, by Van Valen [76], and one which has inspired many evolutionary theories that consider biotic interactions as significant mechanisms for evolutionary change [77]. Competitive environmental interactions displayed by hosts and parasites result in a cycle of continuous variation and selection featuring adaptation of the host and counter-adaptations by the parasite, and this mechanism is extremely pronounced in phage-microbe interactions [75]. However, biological evolution has limitations by its very nature and the mechanisms it uses, such as fitness, natural selection, mutation rate, etc. For each stage of progress and evolutionary improvement, the evolving organism pays a price. For example, a high mutation rate leads to increased variance and helps in adapting to an environment, but although frequent mutations in a viral tail fibre can help in colonising new niches with another host receptor faster, the same feature can lead to lower genome stability, decreasing the efficiency of viral proteins and overall fitness.

In the competition with other phages and the ‘arms race’ with the host-parasite, jumbo phages seem to have collected various adaptation genes in their genome, including those that can help to avoid infection from other phages and complement bacterial anti-phage defense systems [3,78]. The analysis of the PaBG genome points to the presence of RM-like and Cas-like proteins. Moreover, the diversity of RM-like proteins in the PaBG genomes suggests the presence of an RM system in PaBG, similar to those shown for other phages [79]. This suggestion can be studied in further research.

Some other proteins encoded in the phage genomes (like amino acid and nucleic acid synthesis related genes) apparently can influence the metabolic processes in the host cell. When considering possible protein functions, one should not rule out the possibility that, in some natural microbial communities, phages are not the only predators in relation to their prey-bacteria, and the complex nature of community interspecies relations might have an impact on the bacterial host. For instance, phages can constrain protist, predation-driven attenuation of *Pseudomonas aeruginosa* virulence in multi-enemy communities, weakening protist-driven, anti-predatory defense (biofilm formation) [80]. The presence of *alg*Z, an alginate and motility regulator gene, in the PaBG genome could, hypothetically, be related with influencing the host in a manner beneficial for the phage.

The strategy of acquiring adaptation genes, frequently pursued by jumbo phages, requires an advanced nucleic acid processing apparatus, and this results in the emergence of a large genome. If we apply this reasoning to the PaBG’s precise replication (including the UDG-domain containing DNAP I) and reparation apparatus, we can suggest that only accurate replication makes the implementation of the strategy possible, diminishing the influence of negative mutations on the adopted genes.

In terms of reproductive strategy, jumbo phages, unlike small lytic phages, appear to adopt a *K*-strategy, rather than an *r*-strategy [81]. A larger genome size and increased structural complexity should lead to greater replication time, increasing the intracellular period of the viral life cycle. The work of advanced replication apparatus and numerous repair enzymes could be considered as a mechanism for ‘caring for the offspring’. The experimental data showed comparatively low progeny from the PaBG (30–40 particles/cell) and a long growth cycle (it took more than 3 h to complete lysis at 25 °C). Phage T7 (with a much smaller genome size, of roughly 40 kbp), often used as a model system, produces approximately 100 progeny per infected cell, within 40 min at 30 °C [82]. Even though some basic *K* and *r* characteristics do not seem applicable to DNA viruses [83], available data indicate that the well-documented trade-off in macro-organisms between offspring production and offspring quality also applies to phages [84]. *K*-strategists among macro-organisms can be more competitive and better adapted to emerging micro-environmental characteristics [85]. We hypothesise that, in the circumstances of a scarce reserve of nutrients in the oligotrophic Lake Baikal ecosystem [7,9], the *K*-strategy chosen by PaBG allows it to exploit the available resources more efficiently. Moreover, based on this thinking, we could expect to find *K*-strategist jumbo-phages in diverse, balanced, complex, competitive multispecies communities.

The findings of the phylogenetic analysis of special group traits, in combination with a wide spectrum of bacterial hosts of Lupandier group members, are testament to the long evolutionary history of this ancient family. The presence of homologous genes of Lupandier phages, and related *Measeafarmviruses* in taxonomically diverse and distant groups of non-viral organisms [86], should be the subject of further studies into this phage family, and this may help shed light on the details of the evolution of the Lupandier jumbo phage group.

## 5. Conclusions

*Pseudomonas* phage PaBG is a representative of a separate group of bacteriophages with specific features and their own evolutionary history, which, together with *Measeafarmviruses*, forms a large, ancient phage group. The analysis of published sequences from diverse habitats suggests that the phages of this group are present in various ecosystems, infecting taxonomically different organisms. Sharing a number of traits with other jumbo phages, PaBG has genomic and structural traits inherent to a separate group, which might be on the level of a genus. This genus also includes *Pseudomonas* phage Lu11; *Trichodesmium* phage NCTB; *Dickeya* phages vB_DsoM_AD1, vB_DsoM_JA13, vB_DsoM_JA29, vB_DsoM_JA11 and vB_DsoM_JA33; and *Erwinia* phages vB_EamM_Y3, vB_EamM_Yoloswag and vB_EamM_Alexandra, as described earlier.

## Figures and Tables

**Table 1:** Protein bands identified in the structural proteome of phage PaBG.

Band	MW, Da	PaBG Gene Product	Putative Function
1	221,161.95	202	RuvB helicase-like protein
2	159,716.54	215	long tail fibre
3	83,518.18	240	base plate wedge subunit protein
4	58,471.68	213	tail sheath protein
5	57,891.91	76	tail sheath protein
6	57,162.97	257	putative tail component
7	54,891.94	94	tail assembly protein
8	35,272.92	191	Unknown
9	35,127.30	83	Unknown
10	34,778.160	261	Unknown
11	40,142.490	80	major capsid protein
12	23,463.300	81	Unknown
13	20,796.47	214	putative tail component
14	18,908.470	263	Unknown

## Supplementary Materials

The following are available online at https://www.mdpi.com/1999-4915/12/7/721/s1, Supplementary Figure S1: Phylogenetic tree generated by VICTOR using the complete genomic sequences of the 15 phage species, including three *Measeafarmvirus* phages, *Pseudomonas* phage PaBG, *Pseudomonas* phage Lu11, Phage NCTB, *Dickeya* phage vB_DsoM_JA29, *Dickeya* phage vB_DsoM_JA13, *Dickeya* phage vB_DsoM_JA11, *Dickeya* phage vB_DsoM_JA33, *Erwinia* phage vB_EamM_Yoloswag, *Erwinia* phage vB_EamM_Y3, *Erwinia* phage vB_EamM_Alexandra, *Dickeya* phage vB_DsoM_AD1 and *Enterobacteria* phage T4. Supplementary Figure S2: Best-scoring tree found by ML search with RAxML based on the major capsid protein sequences. Taxonomic classification is taken from NCBI sequence attributes and is shown to the right of the organism name. Bootstrap support values are shown above their branch as a percentage of 1000 replicates. The scale bar shows 0.5 estimated substitutions per site and the tree was unrooted. Supplementary Figure S3: Best-scoring tree found by ML search with RAxML based on the terminase large subunit protein sequences. Taxonomic classification is taken from NCBI sequence attributes and is shown to the right of the organism name. Bootstrap support values are shown above their branch as a percentage of 1000 replicates. The scale bar shows 0.5 estimated substitutions per site and the tree was unrooted. Supplementary Figure S4: Best-scoring tree found by ML search with RAxML based on the major capsid protein and terminase large subunit concatenated protein sequences. Taxonomic classification is taken from NCBI sequence attributes and is shown to the right of the organism name. Bootstrap support values are shown above their branch as a percentage of 1000 replicates. The scale bar shows 0.5 estimated substitutions per site and the tree was unrooted. Supplementary Figure S5: Phylogenetic tree obtained with MrBayes based on the metagenomic and environmental protein sequences homologous to the PaBG major capsid protein. Bayesian posterior probabilities are indicated above their branch. Taxonomic classification is taken from NCBI sequence attributes and is shown to the right of the organism name. The scale bar shows 0.1 estimated substitutions per site and the tree was rooted to *Bacillus* virus *G*; 3,000,000 generations sampled every 100 generations and an average standard deviation of split frequencies of 0.0094. Supplementary Figure S6: Genome sequence comparison among *Salmonella enterica* subsp. *enterica* serovar Infanti*s* strain 159669 and five PaBG-related viral genomes exhibiting co-linearity detected by TBLASTX. Phage abbreviations are as follows: Motura, *Achromobacter* phage Motura (*Mieseafarmvirus*); PaBG, *Pseudomonas* phage PaBG; Lu11, *Pseudomonas* phage Lu11; NCTB, Phage NCTB; JA29, *Dickeya* phage vB_DsoM_JA29; AD1, *Dickeya* phage vB_DsoM_AD1; SI, *Salmonella enterica subsp. enterica serovar Infantis* strain 159669. The percentage of sequence similarity is indicated by the intensity of the grey colour. Vertical blocks between analysed sequences indicate regions with at least 16% similarity. Supplementary Figure S7: Bootstrap consensus tree found by ML search with RAxML based on the DNA polymerase I protein sequences extracted from phage GenBank annotations and homologous sequences found with a BLAST search on NCBI databases. Taxonomic classification is taken from NCBI sequence attributes and is shown to the right of the organism name. Bootstrap support values are shown above their branch as a percentage of 1000 replicates. The scale bar shows 0.5 estimated substitutions per site and the tree was rooted to *Bacillus* virus *G*. Supplementary Figure S8: 3D structure homology modelling of the DNA polymerase I and 5′→3′ exonuclease (PaBG gp 205 and gp 137, correspondingly) made with Phyre2. 80% of residues modelled at >90% confidence. (I) Final structure of region included residues 33-933. Uracil-DNA glycosylase domain is highlighted with a circle. (II) 3D-matching of modelled uracil-DNA glycosylase domain (confidence 100%, coloured blue) with best-fitting model (PDB entry 1ui0a of crystal structure of uracil-DNA glycosylase from *Thermus thermophilus* hb8, coloured pale) made with UCSF Chimera. (III) 3D structure homology modelling of the DNA polymerase I and 5′→3′ exonuclease (confidence 96.7%). The models are coloured based on a rainbow gradient scheme, where the N-terminus of the polypeptide chain is coloured blue and the C-terminus is coloured red. Supplementary Figure S9: Phylogenetic tree obtained with MrBayes based on the 5′→3′ exonuclease protein sequences and homologous sequences found with a BLAST search on NCBI databases. Bayesian posterior probabilities are indicated above their branch. Taxonomic classification is taken from NCBI sequence attributes and is shown to the right of the organism name. The scale bar shows 0.2 estimated substitutions per site and the tree was rooted to sequence *Bacillus* virus *G*; 3,000,000 generations sampled every 100 generations and an average standard deviation of split frequencies of 0.0240. Supplementary Figure S10: (I) 3D structure homology modelling of the DNA polymerase III subunit β (PaBG gp 207) made with Phyre2. 97% of residues were modelled at 100% confidence. (II) 3D structure homology modelling of the DNA polymerase III subunit γ made with Phyre2. 91% of residues were modelled at 100% confidence. The models are coloured based on a rainbow gradient scheme, where the N-terminus of the polypeptide chain is coloured blue and the C-terminus is coloured red. Supplementary Figure S11: Phylogenetic tree obtained with MrBayes based on the DNA polymerase III subunit β protein sequences and homologous sequences found with a BLAST search on NCBI databases. Bayesian posterior probabilities are indicated above their branch. Taxonomic classification is taken from NCBI sequence attributes and is shown to the right of the organism name. The scale bar shows 0.1 estimated substitutions per site and the tree was rooted to *Corynebacterium* phage Colleen; 3,000,000 generations sampled every 100 generations and an average standard deviation of split frequencies of 0.0532. Supplementary Figure S12: Phylogenetic tree obtained with MrBayes based on the RNA polymerase σ70 factor protein sequences and homologous sequences found with a BLAST search on NCBI databases. Bayesian posterior probabilities are indicated above their branch. Taxonomic classification is taken from NCBI sequence attributes and is shown to the right of the organism name. The scale bar shows 0.2 estimated substitutions per site and the tree was rooted to *Bacillus* virus *G*; 3,000,000 generations sampled every 100 generations and an average standard deviation of split frequencies of 0.0084. Supplementary Figure S13: 3D structure homology modelling of the PaBG major capsid protein (gp80). (I) Final structure obtained by HHpred (https://toolkit.tuebingen.mpg.de/tools/hhpred). The models are coloured based on a rainbow gradient scheme, where the N-terminus of the polypeptide chain is coloured blue and the C-terminus is coloured red. (II) 3D-matching of obtained PaBG major capsid protein model (coloured blue) with best-fitting model (PDB entry 3JB5 of crystal structure of major capsid protein from *Propionibacterium acnes Bacteriophage ATCC*, coloured pale brown) made with UCSF Chimera. Supplementary Figure S14: Phylogenetic tree obtained with MrBayes based on the major capsid protein sequences and homologous sequences found with a BLAST search on NCBI databases. Bayesian posterior probabilities are indicated above their branch. Taxonomic classification is taken from NCBI sequence attributes and is shown to the right of the organism name. The scale bar shows 0.2 estimated substitutions per site and the tree was rooted to *Bacillus* virus *G*; 3,000,000 generations sampled every 100 generations and an average standard deviation of split frequencies of 0.0080. Supplementary Figure S15: Phylogenetic tree obtained with MrBayes based on the terminase large subunit protein sequences and homologous sequences found with a BLAST search on NCBI databases. Bayesian posterior probabilities are indicated above their branch. Taxonomic classification is taken from NCBI sequence attributes and is shown to the right of the organism name. The scale bar shows 0.2 estimated substitutions per site and the tree was rooted to *Bacillus* virus *G*; 3,000,000 generations sampled every 100 generations and an average standard deviation of split frequencies of 0.0033. Supplementary Figure S16: MAFFT alignment of the terminase large subunit (L-INS-i algorithm, BLOSUM62 scoring matrix, 1.53 gap open penalty, 0.123 offset value). Intein domains found with InterPro are shown with green lanes. Vertical black lanes indicate similarity between sequences. Supplementary Table S1: Infectious range of PaBG. Supplementary Table S2: Functional assignments of *Pseudomonas* phage PaBG genes. Supplementary Table S3: Average nucleotide identity (ANI) between *Pseudomonas* phage PaBG and all 12,207 phage genomes deposited to NCBI GenBank (up to February 2020) with OrthoANIu with default settings. Supplementary File S1: The genome of *Pseudomonas* phage PaBG in GenBank format. Supplementary File S2: Sequences, alignments, trees.
