# Supplementary material for: Pseudomonas Phage PaBG—A Jumbo Member of an Old Parasite Family
Source: Viruses. 2020 Jul 3;12(7):721. doi: 10.3390/v12070721 (PMC7412058; doi:10.3390/v12070721)
Supplement: Supplementary file 1 [file viruses-12-00721-s001.zip › Supplementary Table 01 - Phage Host Range Determination2.docx]

**Table 1**. Host range of bacteriophage PaBG on strains *Pseudomonas aeruginosa* as determened by spot testing

| *P. aeruginosa* from collection of State Research Center for Applied Biotechnology and Microbiology (FBIS SRCAMB), Obolensk | strains | Bacteriophage PaBG |
| --- | --- | --- |
|  | 99 | + |
|  | 513 | + |
|  | 704 | - |
|  | 214 | - |
|  | 227 | - |
|  | 235 | - |
|  | 239 | - |
|  | 255 | - |
|  | 256 | - |
|  | 266 | - |
|  | 282 | - |
|  | 310 | - |
|  | 326 | - |
|  | 379 | - |
|  | 382 | - |
|  | 394 | - |
|  | 413 | - |
|  | 453 | - |
|  | 468 | - |
|  | 469 | - |
|  | 479 | - |
|  | 489 | - |
|  | 512 | - |
|  | 515 | - |
|  | 520 | - |
|  | 521 | - |
|  | 3004 | - |
|  | 3008 | - |
|  | 3019 | - |
|  | 3027 | - |
|  | 3043 | - |
|  | 3055 | - |
|  | 3068 | - |
| *P. aeruginosa* from collection of Institute of Chemical Biology and Fundamental Medicine (ICBFM SB RAS), Novosibirsk |  |  |
|  | 38 | - |
|  | 662 | + |
|  | 663 | - |
|  | 664 | - |
|  | 665 | - |
|  | 666 | - |
|  | 667 | - |
|  | 668 | - |
|  | 669 | + |
|  | 670 | + |
|  | 671 | - |
|  | 712 | - |
|  | 1097 | - |
|  | 1152 | - |
|  | 1654 | - |
|  | 1655 | - |
|  | 1693 | - |
|  | 1697 | - |
|  | 1699 | - |
|  | 1710 | - |
|  | 1711 | - |
|  | 1725 | + |
|  | 1729 | - |
|  | 1758 | - |
|  | 1759 | - |
|  | 1760 | - |
|  | 1764 | - |
|  | 1778 | - |
|  | 1781 | - |
|  | 1783 | - |
|  | 1804 | - |
|  | 1805 | - |
|  | 1806 | - |
|  | 1816 | - |
|  | 1817 | - |
|  | 1818 | - |
|  | 1823 | - |
|  | 1824 | - |
|  | 1828 | - |
|  | 1836 | - |
|  | 1978 | - |
|  | 1979 | - |
|  | 1980 | - |
|  | 1981 | - |
|  | 2037 | - |
|  | 2052 | - |
|  | 2065 | - |
|  | 2068 | - |
|  | 2075 | - |
|  | 2080 | - |
|  | 2102 | - |
|  | 2106 | - |
| *P. aeruginosa* from collection of bacteriophage genetics laboratory of prof. Krylov V.N. of Federal Budget Institution of Science Mechnikov Vaccine and Serum Research Institute (FBIS VSRI) |  |  |
|  | PAO | + |
|  | ELR2 | + |
|  | Sch | - |
|  | 1-14 | + |
|  | 2-10 | + |
|  | 5-2 | + |
|  | 7-6 | - |
|  | 7-10 | - |
|  | 7-25 | + |
|  | 8-14 | - |
|  | 8-20 | + |
|  | 9-6 | - |
|  | 9-11 | + |
|  | Che 1 | - |
|  | Che 4 | - |
|  | Che19/6 | + |
|  | Che26/6 | + |
|  | Che 31/6 | - |
|  | 2001 | + |
|  | 2002 | - |
|  | 2003 | - |
|  | 2004 | - |
|  | 2005 | + |
|  | 2006 | + |
|  | 2007 | + |
|  | 2008 | - |
|  | 2009 | - |
|  | 2010 | - |
|  | Pu21(PMG1) | - |
|  | Pu21(RMS165 | - |
|  | P38(PMG35) | - |
|  | Pu21(RMS149) | - |
|  | P38(PMT53) | - |
|  | P8(PMG70) | - |
|  | P38(PMG671) | - |
|  | P38(R151) | - |
|  | Pse163(B44b) | - |
|  | Pse164 | - |
|  | 22 | - |
|  | 73 | - |

A total of 125 non-duplicate (one per patient) multidrug-resistant (MDR) nosocomial *P aeruginosa* isolates, selected to represent diverse geographical origins were used to assess the spectra of lytic activity of PaBG phage. The studies of the spectrum of lytic activity of phage were carried out in Federal Budget

Institution of Science State Research Center for Applied Biotechnology and Microbiology (FBIS SRCAMB) by Shishkova N.A. , in the bacteriophage genetics laboratory of professo Krylov V.N.of Federal Budget Institution of Science Mechnikov Vaccine and Serum Research Institute (FBIS VSRI) by Pleteneva E.A. and in the laboratory of molecular biology of Institute of Chemical Biology and Fundamental Medicine (ICBFM SB RAS) by Morozova V.

Only 2 *P. aeruginosa* strains (99 and 513) of 33 tested strains (99, 704, 214, 227, 235, 239, 255, 256, 266, 282, 310, 326, 379, 382, 394, 413, 453, 468, 469, 479, 489, 512, 513, 515, 520, 521, 3004, 3008, 3019, 3027, 3043, 3055, 3068) from the FBIS SRCAMB collection were sensitive to bacteriophage PaBG.

40 strains from collection of professor V.N.Krylov (the bacteriophage genetic laboratory of FRIS VSRI) were tested

: PAO, ELR2, Sch, 7-25, 2-10, 8-20, 7-6, 1-14, 5-2, 9-6, 8-14, 9-11, Che 1, Che 4, Che19/6, Che26/6, Che 31/6, 2001, 2002, 2003, 2004, 2005, 2006, 2007, 2008, 2009, 2010, Pu21(PMG1), Pu21(RMS165), P38(PMG35), Pu21(RMS149), P38(PMT53}, P8(PMG70), P38(PMG671), P38(R151), Pse156, Pse163(B44b), Pse164, 22, 73. PaBG phage lysed 15 strains: PAO, ELR2, 7-25, 2-10, 8-20, 1-14, 5-2, 9-11, Che 19/6, Che 26/6, 2001, 2005, 2006, 2007.

53 MDR P. aeruginosa strains tested in the laboratory of molecular biology ICBFM SB RAS:

38, 662, 663, 664, 665*, 666*, 667, 668, 669, 670, 671, 712, 1097, 1152, 1654, 1655, 1693, 1697, 1699, 1710, 1711, 1725, 1729, 1758, 1759, 1760, 1764, 1778, 1781, 1783, 1804, 1805, 1806, 1816, 1817, 1818, 1823, 1824, 1828, 1836, 1978, 1979, 1980, 1981, 1982, 2037, 2052, 2065, 2068, 2075, 2080, 2102, 2106. Only 4 *P. aeruginosa* strains (662, 669, 670, 1725 were sensitive to bacteriophage PaBG.
