# Supplementary figures and images for: Pseudomonas Phage PaBG—A Jumbo Member of an Old Parasite Family
Source: Viruses. 2020 Jul 3;12(7):721. doi: 10.3390/v12070721 (PMC7412058; doi:10.3390/v12070721)

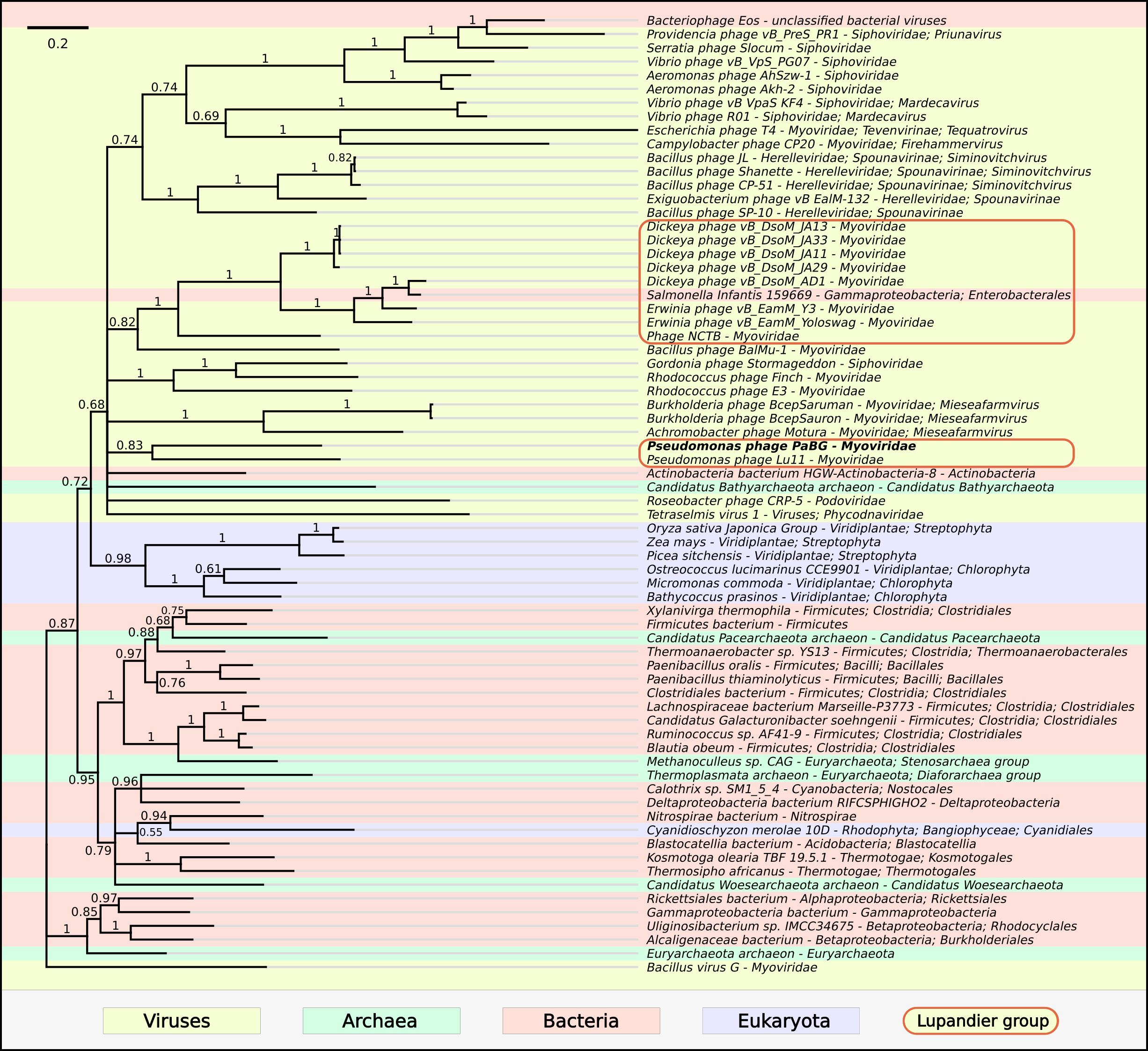

Supplement: Supplementary file 1 [file viruses-12-00721-s001.zip › Supplementary Figure 09 - Tree_5-3_exonuclease.png]

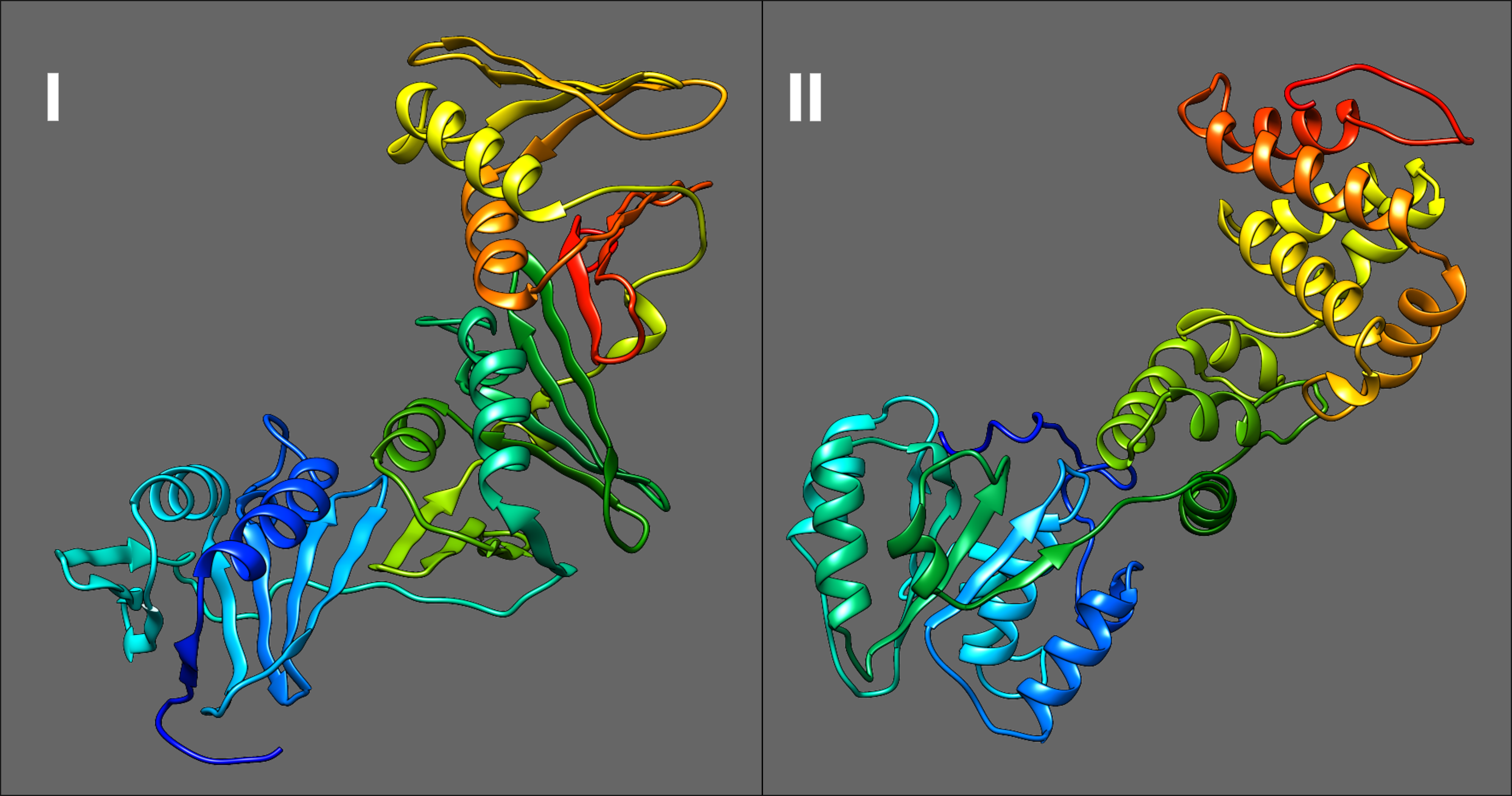

Supplement: Supplementary file 1 [file viruses-12-00721-s001.zip › Supplementary Figure 10 - 3D-DNAP-III.png]

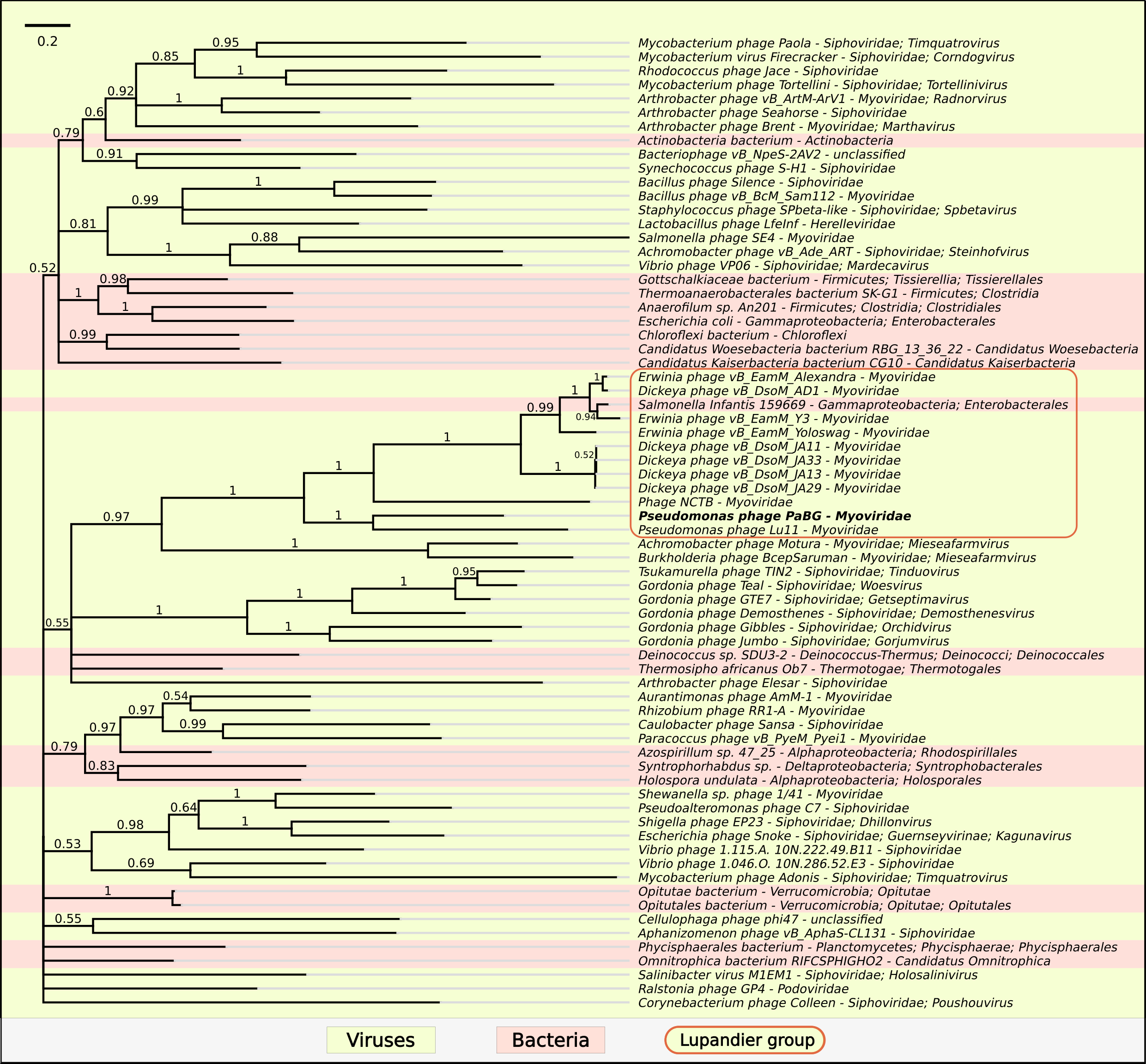

Supplement: Supplementary file 1 [file viruses-12-00721-s001.zip › Supplementary Figure 11 - Tree_DNAP-III-beta.png]

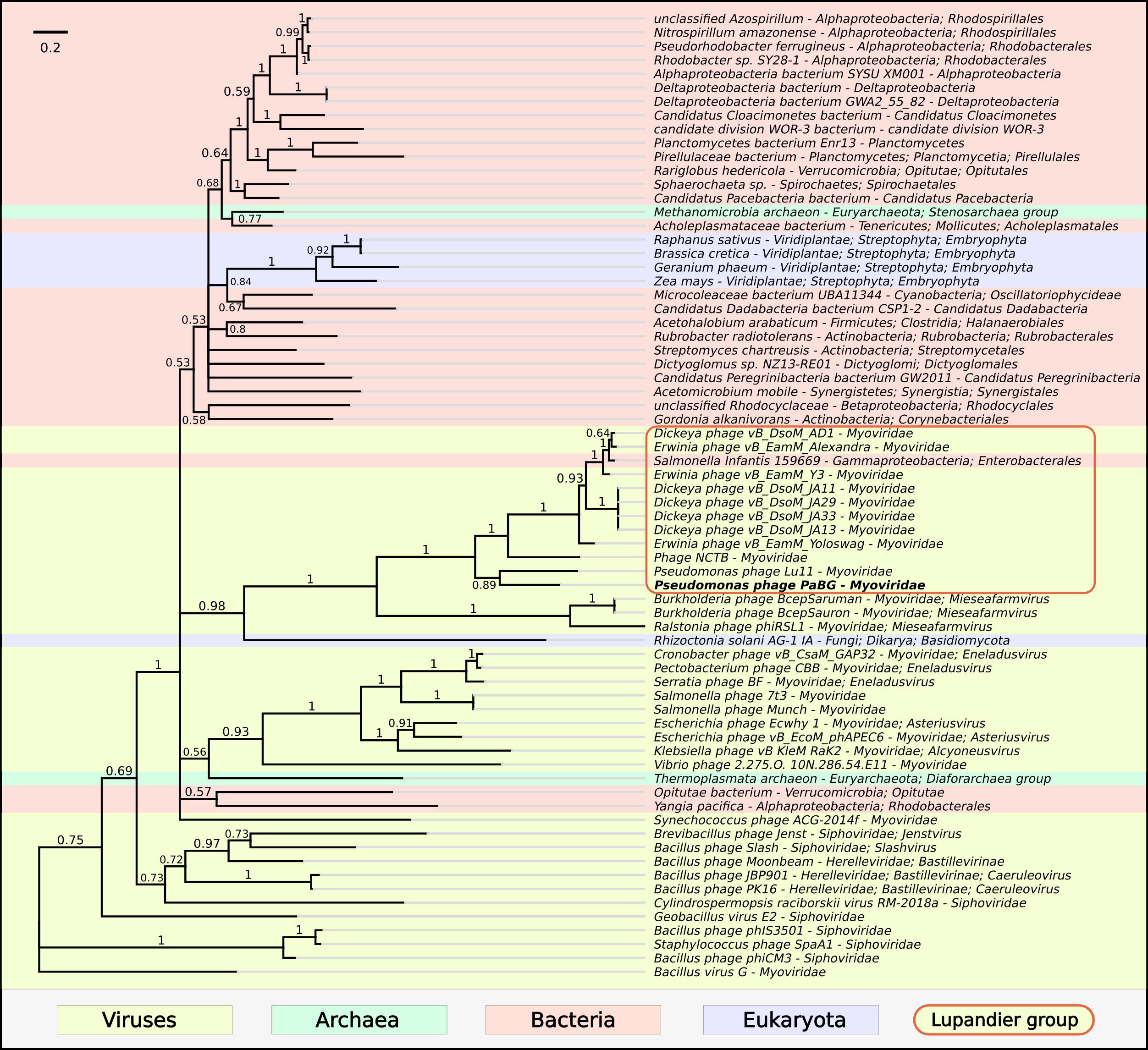

Supplement: Supplementary file 1 [file viruses-12-00721-s001.zip › Supplementary Figure 12 - Tree_Sigma-factor.png]

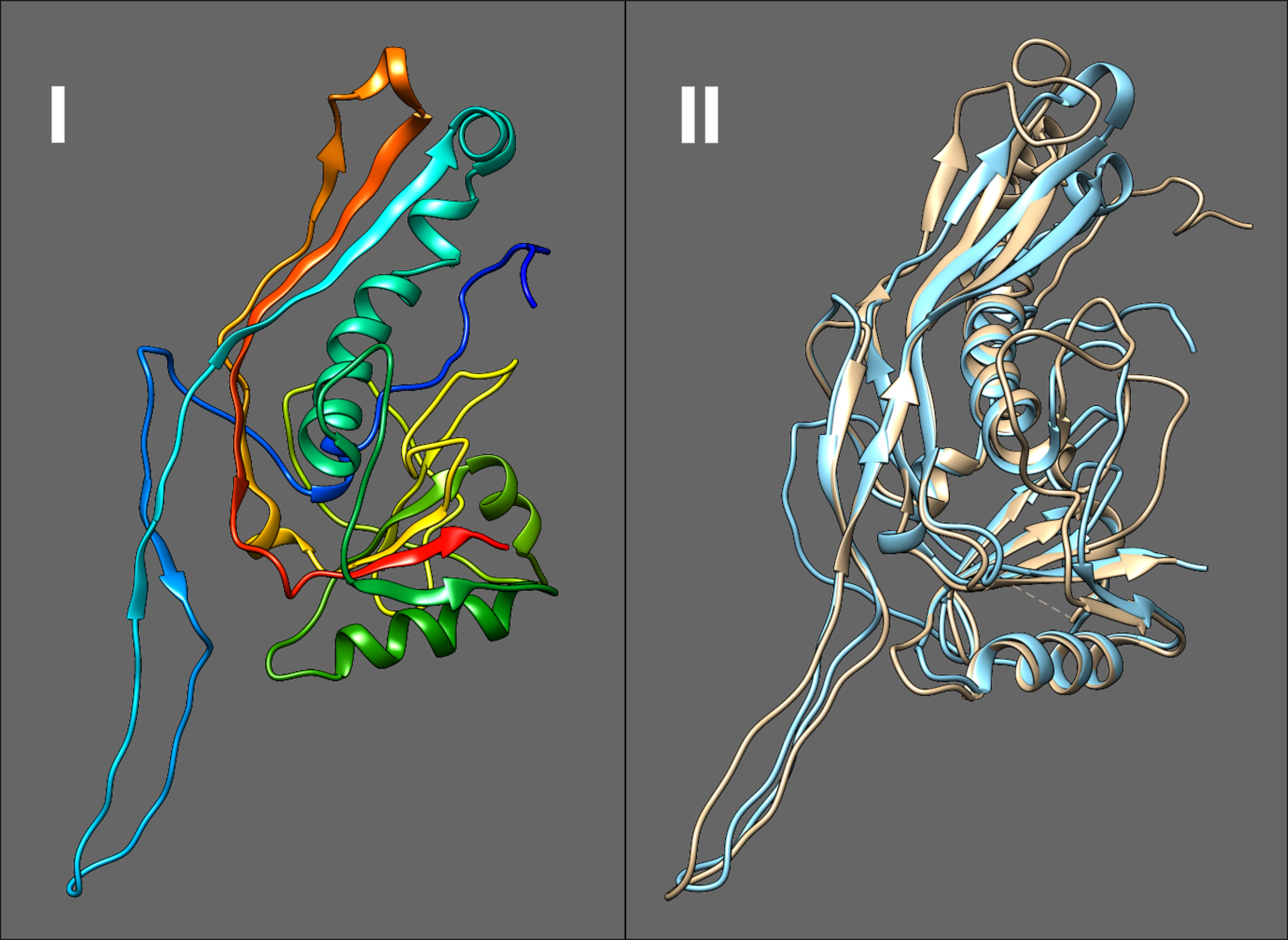

Supplement: Supplementary file 1 [file viruses-12-00721-s001.zip › Supplementary Figure 13 - 3D_MCP.png]

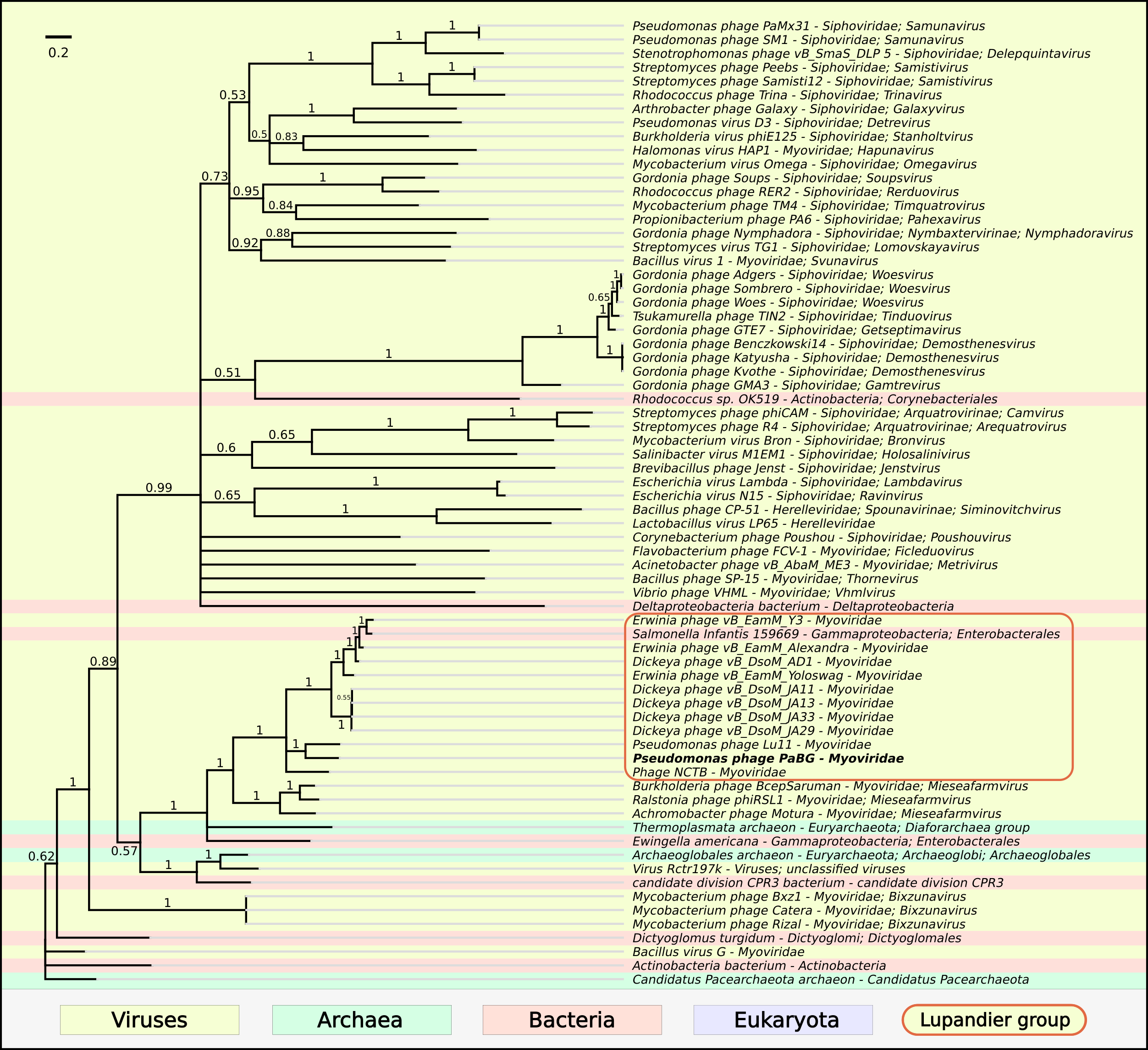

Supplement: Supplementary file 1 [file viruses-12-00721-s001.zip › Supplementary Figure 14 - Tree_MCP.png]

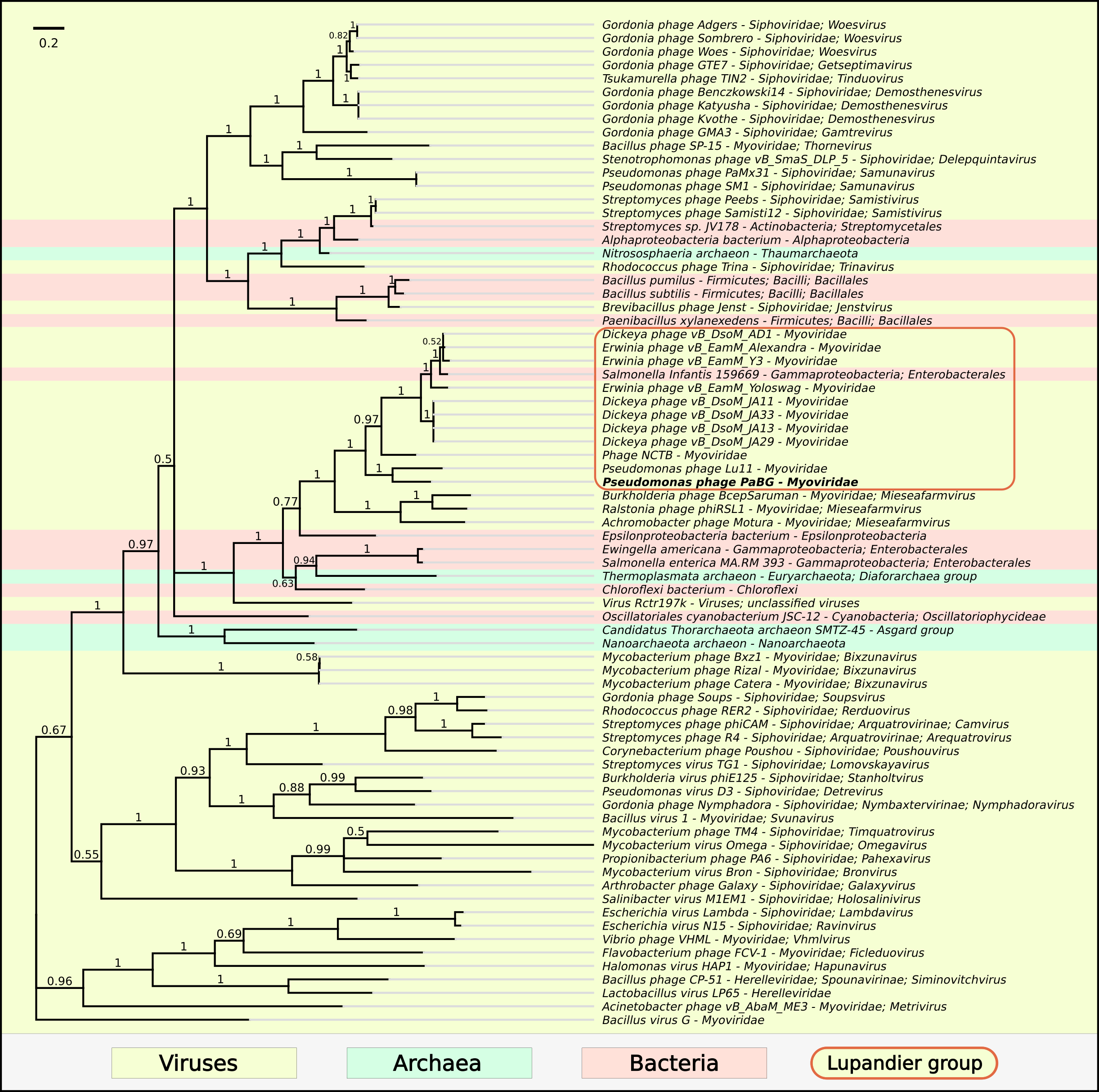

Supplement: Supplementary file 1 [file viruses-12-00721-s001.zip › Supplementary Figure 15 - Tree_TerL.png]

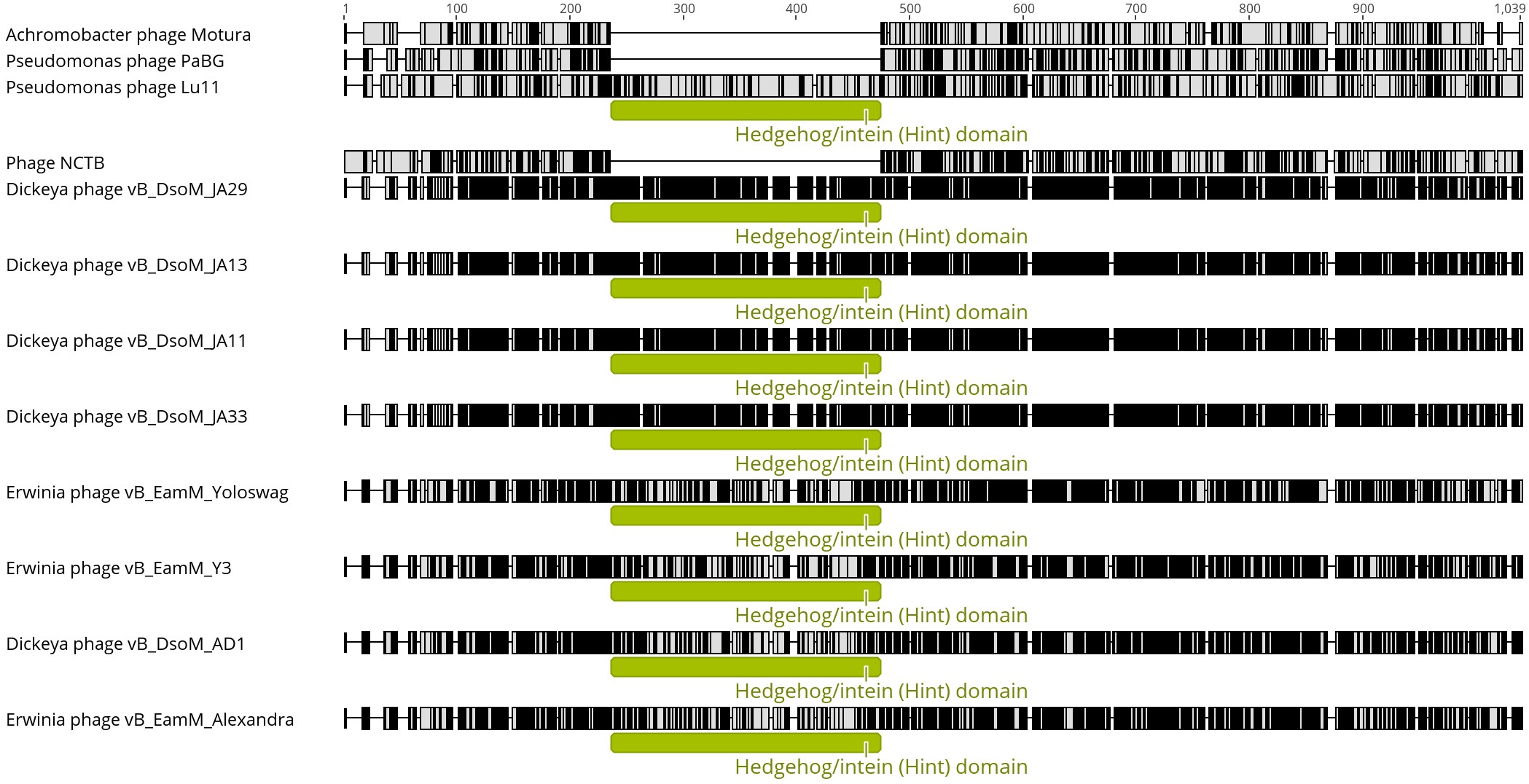

Supplement: Supplementary file 1 [file viruses-12-00721-s001.zip › Supplementary Figure 16 - Terminase_inteins.jpg]

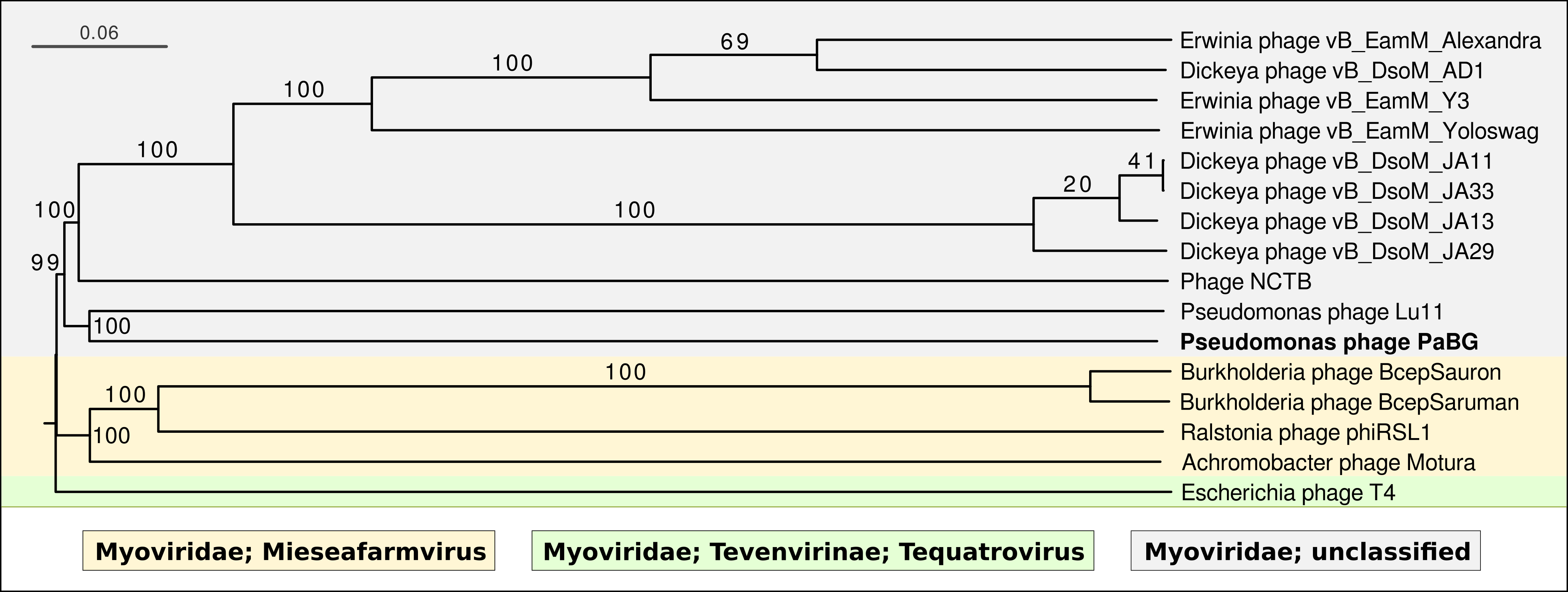

Supplement: Supplementary file 1 [file viruses-12-00721-s001.zip › Supplementary Figure 01 - VICTOR-phylogeny.png]

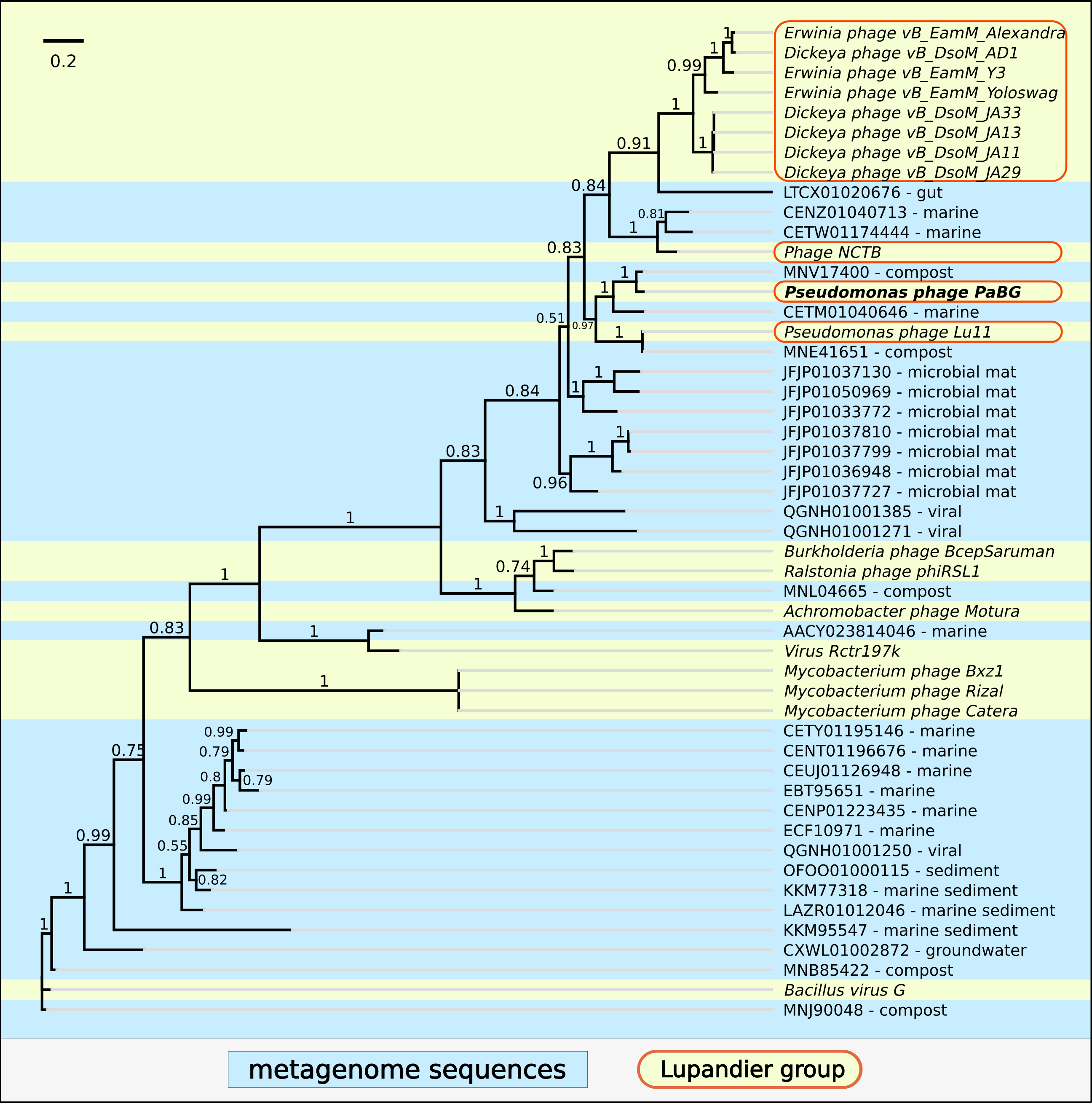

Supplement: Supplementary file 1 [file viruses-12-00721-s001.zip › Supplementary Figure 05 - Tree_environmental_sequences.png]

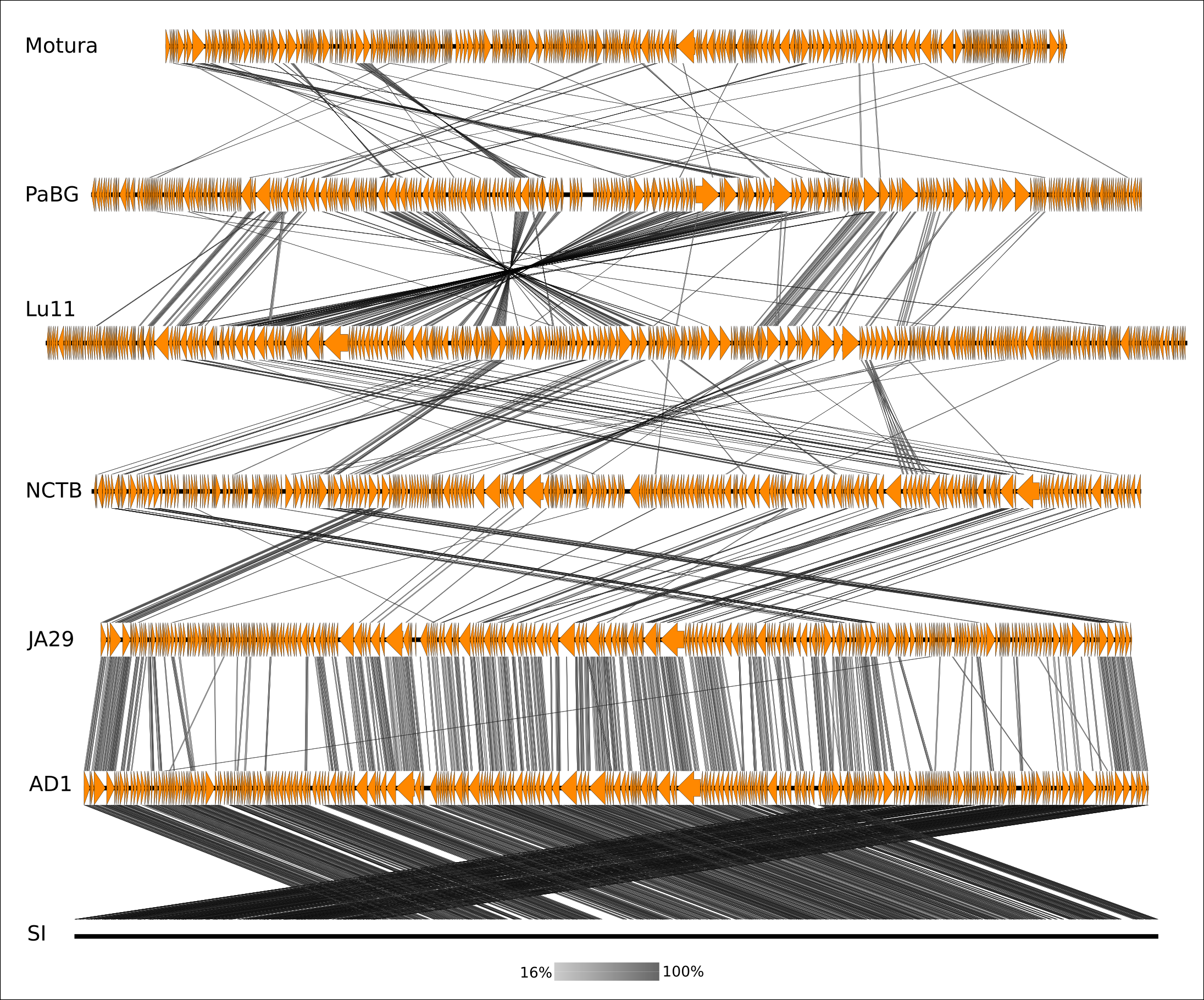

Supplement: Supplementary file 1 [file viruses-12-00721-s001.zip › Supplementary Figure 06 - SI_genome_comparison.png]

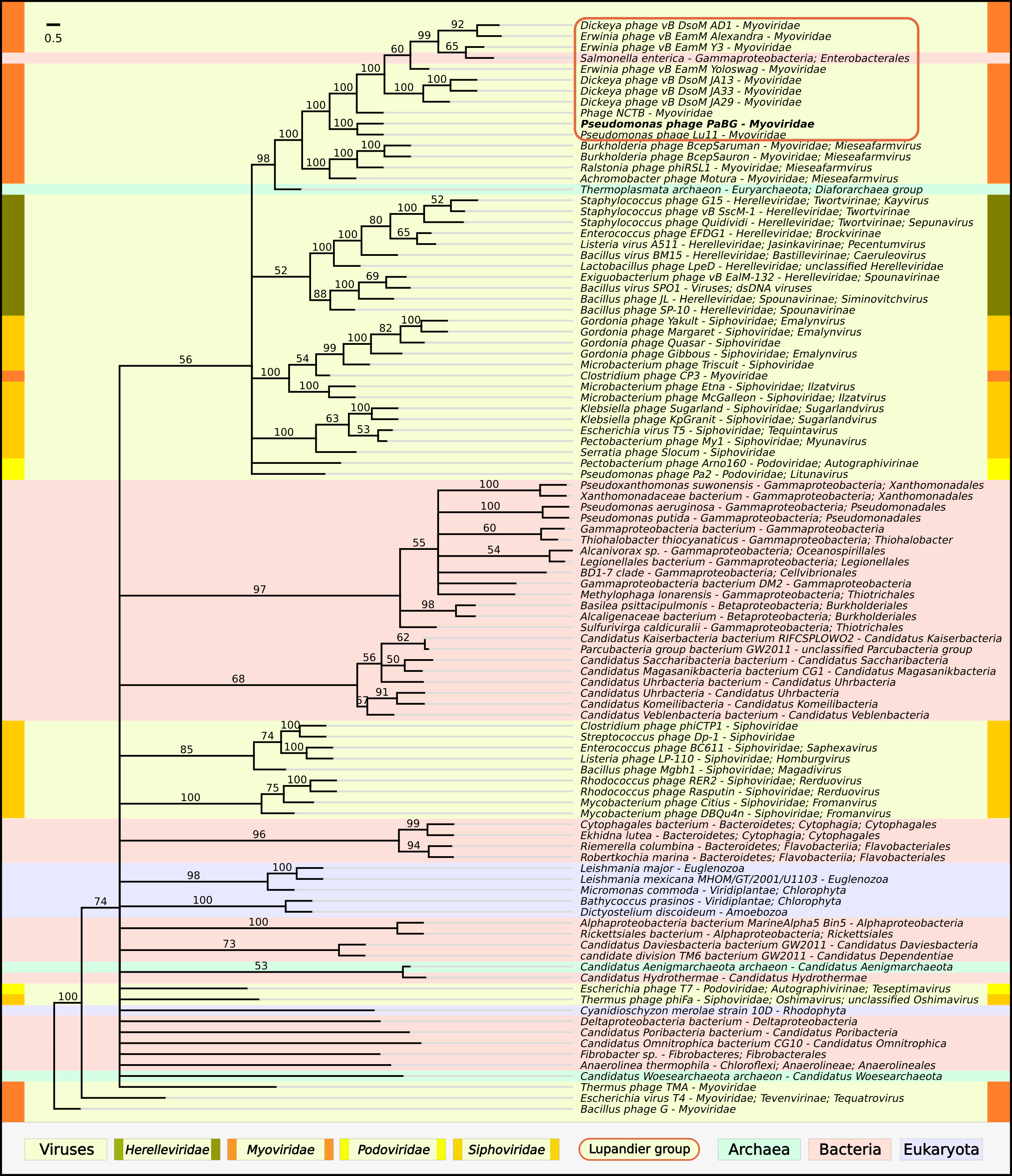

Supplement: Supplementary file 1 [file viruses-12-00721-s001.zip › Supplementary Figure 07 - Tree_DNAP-I.png]

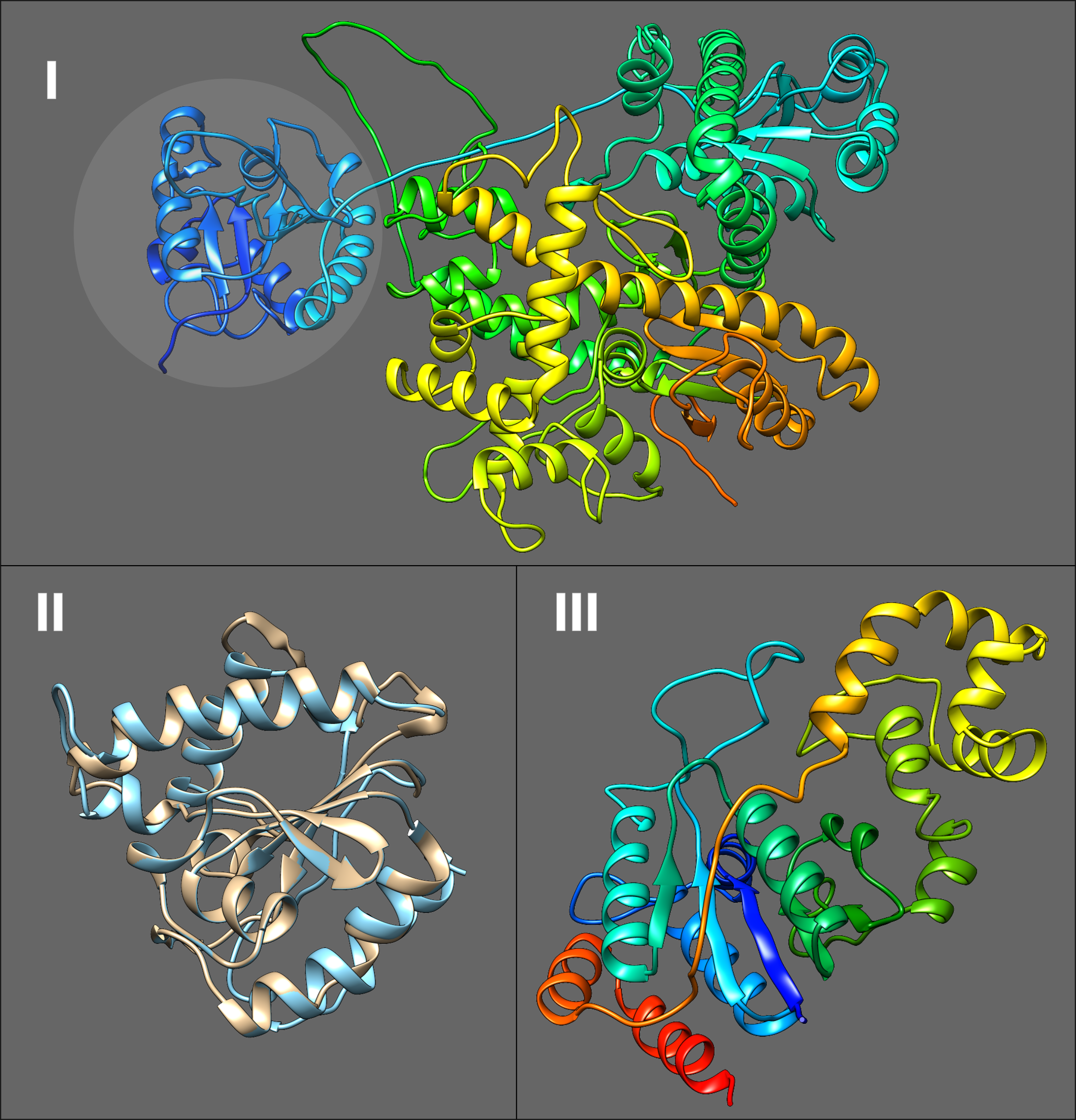

Supplement: Supplementary file 1 [file viruses-12-00721-s001.zip › Supplementary Figure 08 - 3D-DNAP-I.png]
